# Designing Hierarchical Soft Network Materials with Developable Lattice Nodes for High Stretchability

**DOI:** 10.1002/advs.202206099

**Published:** 2023-01-25

**Authors:** Jianxing Liu, Haoyu Guo, Haiyang Liu, Tongqing Lu

**Affiliations:** ^1^ State Key Lab for Strength and Vibration of Mechanical Structures Soft Machines Lab Department of Engineering Mechanics Xi'an Jiaotong University Xi'an 710049 P. R. China

**Keywords:** constituent material, hierarchical‐inspired design, lattice node, soft network material, stretchability

## Abstract

Soft network materials (SNMs) represent one of the best candidates for the substrates and the encapsulation layers of stretchable inorganic electronics, because they are capable of precisely customizing the J‐shaped stress–strain curves of biological tissues. Although a variety of microstructures and topologies have been exploited to adjust the nonlinear stress–strain responses of SNMs, the stretchability of most SNMs is hard to exceed 100%. Designing novel high‐strength SNMs with much larger stretchability (e.g., >200%) than existing SNMs and conventional elastomers remains a challenge. This paper develops a class of hierarchical soft network materials (HSNMs) with developable lattice nodes, which can significantly improve the stretchability of SNMs without any loss of strength. The effects of geometric parameters, lattice topologies, and loading directions on the mechanical properties of HSNMs are systematically discussed by experiments and numerical simulations. The proposed node design strategy for SNMs is also proved to be widely applicable to different constituent materials, including polymers and metals.

## Graphical abstract

This paper proposes a class of hierarchical‐inspired soft network materials (HSNMs) with developable lattice nodes. HSNMs can significantly improve the stretchability of soft network materials without any loss of tensile strength, which is of great importance in practical applications such as stretchable electronics. Mechanical properties of HSNMs can be adjusted by geometric parameter, lattice topology, loading direction, and constituent material.

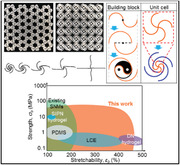

## 1 Introduction

Stretchable inorganic electronics have received extensive attentions in the past decade, owing to the capabilities of offering simultaneously high electrical performances and large deformations.^[^ ^1^, ^2^, ^3^, ^4^, ^5^, ^6^ ^]^ Due to the dramatic advantages, stretchable inorganic electronics have been exploited in a broad range of emerging applications, spreading from long‐term health monitoring and accurate disease therapy,^[^ ^7^, ^8^, ^9^, ^10^, ^11^, ^12^ ^]^ to soft robots and flexible displays.^[^ ^13^, ^14^, ^15^, ^16^, ^17^, ^18^, ^19^, ^20^ ^]^ The stretchability of a device system relies not only on the structure of interconnects between the rigid inorganic electronic components, but also on the properties of substrate and encapsulation layer.^[^ ^21^, ^22^, ^23^, ^24^ ^]^ To achieve a high degree of stretchability, various artificial soft materials (e.g., elastomer, hydrogel) have been employed as the substrate and/or encapsulation layer of the stretchable inorganic electronics.^[^ ^25^, ^26^, ^27^, ^28^, ^29^ ^]^ Although plentiful research efforts have been made to improve the ultimate mechanical properties (e.g., stretchability, strength, and fracture toughness) of these soft materials,^[^ ^30^, ^31^, ^32^, ^33^, ^34^, ^35^, ^36^ ^]^ accessing certain mechanical responses accurately on demand can still be a grand challenge in the field of artificial soft materials.^[^ ^37^ ^]^ In the past few years, soft network materials (SNMs), a class of soft materials with periodically distributed wavy microstructures, have been developed.^[^ ^38^ ^]^ Distinct from the solid soft elastomers which have been widely used before in stretchable inorganic electronics, SNMs are capable of precisely reproducing the J‐shaped stress–strain curves of different human tissues, mainly attribute to the regular lattice‐based constructions.^[^ ^39^, ^40^, ^41^ ^]^ Moreover, imperfection‐insensitivity and high air permeability of SNMs make it possible to improve the function density of the device systems, which is of great concern in practical applications.^[^ ^21^, ^42^, ^43^, ^44^ ^]^

A common approach to construct SNMs is connecting point‐shaped nodes of lattice structures with wavy filamentary microstructures, and plenty of design strategies of microstructures have been proposed for SNMs to realize biomimetic stress–strain responses under large deformations. For example, a representative type of horseshoe‐shaped microstructure has been proposed by Jang et al. for building SNMs with triangular lattice topologies.^[^ ^45^ ^]^ The relationship between the stress–strain responses of these SNMs and the geometries of horseshoe microstructures has been established theoretically.^[^ ^46^, ^47^, ^48^ ^]^ However, most of the existing studies focus only on the mechanical responses before fracture, the ultimate stretchability and tensile strength of SNMs have received far less attention, despite the ultimate mechanical properties are of critical importance for practical applications of SNMs. For example, the triangular SNMs consisting of horseshoe microstructures mentioned above can only offer limited stretchabilities (e.g., <80%), due to the geometric limitations of adjacent microstructures in network structures. Although a variety of other wavy microstructures (e.g., zigzag, crescent, and sinusoid) have been exploited to offer unusual mechanical properties (e.g., negative Poisson's ratios),^[^ ^49^, ^50^, ^51^, ^52^ ^]^ they have done little to improve the stretchability of SNMs (e.g., <150%). The fractal‐inspired strategy which is originally proposed for designing interconnects of stretchable electronics can be used to improve the stretchability of SNMs,^[^ ^53^, ^54^ ^]^ nevertheless, it results in a considerable reduction (e.g., several orders of magnitude) of tensile strength. Note that how to overcome the classical trade‐off between stretchability and tensile strength has plagued the design of soft materials for a long time, and designing SNMs with a combination of large stretchability and high strength remains a challenge. Recently, we attempted to address this issue by proposing a type of SNMs with rotatable lattice nodes.^[^ ^55^ ^]^ However, the design space of these SNMs is restricted by the specific shape of lattice nodes (i.e., circular nodes popular in chiral structures^[^ ^56^, ^57^, ^58^ ^]^), which leads to unavoidable self‐overlay and stress concentration around the nodes in the practical fabrication, thereby resulting in a limited improvement to the stretchability (e.g., <200%) of SNMs.

Besides the traditional approach to design SNMs from the point of wavy filamentary microstructures, lattice nodes can be regarded as another effective route for designing SNMs with additional deformability, which has not been well developed so far. Here, we introduce a unique, hierarchical‐inspired design strategy to significantly improve the stretchability of SNMs by replacing traditional point‐shaped lattice nodes with a class of developable nodes, and the novel SNMs are referred to as hierarchical soft network materials (HSNMs). The building‐block structures of developable nodes are inspired by the Ying–Yang symbol (i.e., Taiji‐diagram) in China, which enable the lattice nodes to be highly stretched. Experimental studies and numerical simulations indicate that the stretchability of SNMs could be improved to over fivefolds (e.g., from ≈50% to ≈250%, for SNMs made of hard polymers) by employing the developable nodes proposed in this work, while ensuring the tensile strength without any reduction. To offer a general design guideline for lattice nodes of SNMs, we further proposed several other types of nodes (e.g., polygonal, starlike, and volute) in addition to the developable nodes. The effect of geometric parameters and lattice topologies on both J‐shaped stress–strain responses and nonlinear Poisson's ratios are systematically analyzed, which allows a clear understanding of the relationship between the macroscopical mechanical properties and the microstructure geometries. The availability of developed design strategy for SNMs made of distinct constituent materials (e.g., hard polymer, brittle resin, and metal) is demonstrated, which suggests the way to a broad range of potential applications, ranging from stretchable electronics to deployable spacecraft structures.

## 2 Results and Discussion

### 2.1 HSNMs with Developable Lattice Nodes

**Figure 1:**
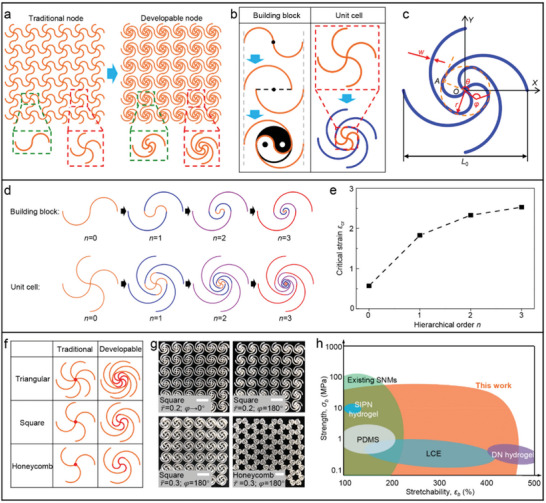
Design concepts of HSNMs with developable nodes. a) Schematic illustration of a HSNM with developable nodes in comparison to a SNM with traditional point‐shaped nodes. The images on the bottom show geometries of the typical building‐block structures (green dashed boxes) and the unit‐cell structures (red dashed boxes) of HSNM and SNM. b) Design strategies for building block and unit cell of proposed HSNMs. The developable nodes are inspired by the Ying–Yang symbol (building block) in China and hierarchical structure (unit cell) in biomaterials. c) Schematic illustration of a typical unit‐cell structure and key geometric parameters of square HSNMs. d) Schematic illustrations of building‐block structures (top) and unit‐cell structures (bottom) for HSNMs with different hierarchical order (*n* = 0, 1, 2, 3). e) Critical strain (*ε*_cr_) versus hierarchical order (*n*) for HSNMs with fixed key geometric parameters (*φ* = 180°, *θ* = 180°, $\bar{r}$ = 0.2 and $\bar{w}$ = 0.01). f) Comparison of unit cells for HSNMs and traditional SNMs with three representative lattice topologies, the lattice nodes are marked in red. g) Optical images of four typical HSNMs fabricated by 3D printing. Apart from the geometric parameters (i.e., $\bar{r}$ and *φ*) marked in the images, the normalized width ($\bar{w}$) and the joint angle (*θ*) are fixed as $\bar{w}$ = 0.04 and *θ* = 180°, respectively. h) Ashby plot of tensile strength (*σ*_b_) versus stretchability (*ε*_b_) for existing SNMs,^[^ ^45^, ^49^, ^50^, ^51^, ^55^ ^]^ typical soft materials (e.g., polydimethylsiloxane (PDMS),^[^ ^59^ ^]^ liquid crystal elastomer (LCE),^[^ ^60^ ^]^ semi‐interpenetrating polymer network (SIPN) hydrogel,^[^ ^61^ ^]^ double network (DN) hydrogel^[^ ^34^ ^]^), and HSNMs proposed in the current work. Scale bars, 10 mm in (g).

**Figure** **1** presents the design methods, representative geometries, and strength‐stretchability characteristics of the HSNMs proposed in this work. As shown in Figure 1a, the proposed HSNMs utilize a class of developable nodes to supersede the traditional point‐shaped nodes which are commonly used in the previous SNMs, and the developable nodes are designed to be composed of a set of circular arcs (e.g., four antisymmetric arcs for HSNMs with square topology). Design concepts of the building‐block structures and the unit‐cell structures of the HSNMs are demonstrated in Figure 1b. The building‐block structures are inspired by the Ying‐Yang diagram in Chinese traditional culture, i.e., a curved filament locating at the dividing line between Ying area and Yang area is employed to connect two offset horseshoe microstructures, as shown in the left panel of Figure 1b. It is noteworthy that the geometry of curved dividing line in Ying–Yang diagram is very similar to that of horseshoe microstructures (i.e., both of them are formed by two identical circular arcs), thereby paving the way to a hierarchical structure. As for unit‐cell structures, take the square HSNMs as an example (right panel of Figure 1b), note that the developable nodes of HSNMs possess the same geometric constructions as the unit cells of traditional SNMs. Therefore, the developable nodes realize a hierarchical structure in SNMs, and the novel SNMs proposed in this work are referred to as hierarchical‐inspired SNMs (i.e., HSNMs).

From a design perspective, the HSNMs represent a brand‐new class of SNMs, which are totally distinct from the traditional SNMs and fractal‐inspired SNMs. The hierarchical structure enables HSNMs to provide superior mechanical properties by means of bring an ingenious deformation mode. Specifically, the developable node design of HSNMs invests the lattice nodes with considerable deformability (e.g., offers additional deformation of nodes in terms of step‐by‐step unfolding and coupled bending), meanwhile, the sectional area of the horseshoe microstructures connected with the lattice nodes are able to be not decreased, thus the proposed HSNMs are capable of improving stretchability without reducing tensile strength. The HSNMs with developable nodes expand the design space of SNMs and provide a much wider accessible range of ultimate mechanical properties for SNMs, which is urgently needed in practical applications such as biointegrated electronics, soft robots, and artificial tissues.

A representative unit cell and key geometric parameters of proposed HSNMs are shown in Figure 1c. To highlight the location and the size of developable node in this unit cell, a dashed circle in orange (with a radius of *r*) is adopted to enclose the developable node. The developable node consists of four identical circular arcs (the arcs located inside the orange dashed circle) with an arc angle of *φ*, and the horseshoe microstructures (i.e., four larger circular arcs outside the orange dashed circle) are connected to the developable node by tangential attaching to the dashed circle at the end of the node arcs (e.g., “*A*” in Figure 1c). The angle between the line *OA* (line from the center (*O*) of the developable node to the end (*A*) of the node arc) and the *X*‐axis is defined as the joint angle *θ*, which can be used to describe the arc angle (*φ*_0_) and the radius (*r*_0_) of horseshoe microstructures (Figure S1, Supporting Information) as,

(1) $$\left\{\begin{array}{c} \varphi_{0}=\theta -\frac{\pi }{2}+\arccos\left[\frac{\left(r-r_{0}\right)\sin\theta }{r_{0}}\right]\\ r_{0}=\sqrt{{\left[\frac{L_{0}}{2}+\left(r_{0}-r\right)\cos\theta \right]}^{2}+{\left[\left(r_{0}-r\right)\sin\theta \right]}^{2}} \end{array}\right.$$

where *L*_0_ is the dimension of unit cell along *X*‐axis. Therefore, four key geometric parameters can be adopted to fully characterize the construction of proposed HSNMs with developable nodes, including the normalized radius ($\bar{r}$ = *r* / *L*_0_), the node angle (*φ*), the joint angle (*θ*), as well as the normalized width ($\bar{w}$ = *w* / *L*_0_) of node arcs and microstructures, as shown in Figure 1c. Noted that the arc angles of developable nodes (*φ*) and microstructures (*φ*_0_) are allowed to be different in a unit cell, which ensures a high degree of design flexibility of the proposed HSNMs. Among these key geometric parameters, the normalized radius ($\bar{r}$), the joint angle (*θ*), and the node angle (*φ*) have obvious influence on the stretchability of HSNMs, while the tensile strength of HSNMs is mainly affected by the normalized width ($\bar{w}$). That is to say, when the normalized width ($\bar{w}$) is fixed, the stretchability of HSNMs can be improved by increasing $\bar{r}$, *φ*, and *θ*, and the tensile strength of HSNMs is almost not decreasing. The tensile strength of HSNMs can be further improved by adopting a higher normalized width ($\bar{w}$).

The design strategy of hierarchical structure and developable node can be extended directly to HSNMs with higher hierarchical order (*n*), as shown in Figure 1d and Figure S2 (Supporting Information). Figure S2c–f presents the deformation sequences of the building‐block structures of HSNMs with different hierarchical orders (*n* = 0, 1, 2, 3) under uniaxial stretching. It can be observed that the building blocks of HSNMs undergo a step‐by‐step unwinding process coupled with the gradual bending deformations of different order of arcs, which is totally distinct from the simple deformation mechanism of the traditional SNMs (i.e., *n* = 0). Note that an increased hierarchical order results in a higher stretchability but a more complex geometry. For example, the developable nodes of square HSNMs with hierarchical order *n* are composed of 4*n* circular arcs, as shown in the bottom panel of Figure 1d. The critical strain (*ε*_cr_, the applied strain to fully extend the building‐block structure) can be used for evaluating the deformability of SNMs, and the critical strain (*ε*_cr_) of HSNMs (*φ* = *θ* = 180°) with different hierarchical order (*n*) can be derived as,

(2) $$\varepsilon_{\mathrm{cr}}=\sum_{i=0}^{n}2\pi i{\bar{r}}^{i}+\frac{\pi }{2}-1$$

Figure 1e provides the effect of hierarchical order (*n*) on the critical strain (*ε*_cr_) of HSNMs with normalized radius fixed as $\bar{r}$ = 0.2. It is noteworthy that the critical strain of HSNMs (*n* > 0) is much larger than that of traditional SNMs (*n* = 0), nevertheless, the critical strain increases more and more slightly as the hierarchical order increases, which indicates a limited improvement of deformability, so the HSNMs with higher hierarchical order (*n* > 1) are not furtherly concerned in this paper. Moreover, it should be pointed out that we focus on the in‐plane deformation of two‐dimensional (2D) HSNMs in this work with the view to serving the practical requirement of mechanical properties in substrate and/or encapsulation layer of the stretchable inorganic electronics, while the out‐of‐plane deformation and 3D structures are out of the scope of the present study.

Apart from the key geometric parameters mentioned above, the mechanical properties of HSNMs also rely on the lattice topologies (i.e., network patterns), which provide one more pathway for material design. Distinct from the point‐shaped nodes in traditional SNMs, the developable nodes of HSNMs possess different geometric constructions in different network topologies, due to the self‐similarity of hierarchical‐inspired structure (Figure 1f). Specifically, a developable node consists of six circular arcs for triangular HSNMs, four circular arcs for square HSNMs, and three circular arcs for honeycomb HSNMs. All of the point‐shaped and developable lattice nodes are marked in red in Figure 1f. The hierarchical order (*n*) of HSNMs with triangular and honeycomb topologies can be also increased by rational design, as shown in Figure S3 (Supporting Information). In addition, the relative density ($\bar{\rho }$, defined as the ratio of volume of cellular material to solid material) can be used to describe the tunable‐porosity attribute of SNMs, and the relative density ($\bar{\rho }$) of HSNMs with developable nodes is given by

(3) $$\left\{\begin{array}{c} {\bar{\rho }}_{\mathrm{triangular}}=4\sqrt{3}\bar{w}\left(\varphi_{0}{\bar{r}}_{0}+\varphi \frac{\bar{r}}{2\sin\left(\varphi /2\right)}\right)\\ {\bar{\rho }}_{\mathrm{square}}=4\bar{w}\left(\varphi_{0}{\bar{r}}_{0}+\varphi \frac{\bar{r}}{2\sin\left(\varphi /2\right)}\right)\\ {\bar{\rho }}_{\mathrm{honeycomb}}=\frac{4\sqrt{3}}{3}\bar{w}\left(\varphi_{0}{\bar{r}}_{0}+\varphi \frac{\bar{r}}{2\sin\left(\varphi /2\right)}\right) \end{array}\right.$$

for the three typical topologies shown in Figure 1f. When the normalized width ($\bar{w}$) and the arc angle (*φ*_0_) of horseshoe microstructures are fixed, the HSNMs with developable nodes are more densely distributed than the SNMs with traditional point‐shaped nodes (i.e., $\bar{r}$ = 0). As a class of artificial architecture materials,^[^ ^62^, ^63^, ^64^, ^65^, ^66^ ^]^ the proposed HSNMs can be manufactured into different scales with diversified constituent materials (e.g., brittle materials, elastic materials, and elastic‐plastic materials), using various advanced techniques. For example, the HSNM specimens with different geometric constructions shown in Figure 1g are made of hard photopolymer (VeroPureWhite, Stratasys) and fabricated by a 3D printer (Object 350, Stratasys). Apart from the 3D printing, the HSNMs can be manufactured using many well‐established planar techniques, such as high‐precision laser cutting and photolithography, attribute to the 2D structure of HSNMs.

Figure 1h presents an Ashby plot that characterizes stretchability (*ε*_b_) and tensile strength (*σ*_b_) of the proposed HSNMs and some other representative soft materials reported previously in the literatures, including the existing SNMs,^[^ ^45^, ^49^, ^50^, ^51^, ^55^ ^]^ polydimethylsiloxane (PDMS),^[^ ^59^ ^]^ liquid crystal elastomers (LCEs),^[^ ^60^ ^]^ semi‐interpenetrating polymer network (SIPN) hydrogels,^[^ ^61^ ^]^ and typical double network (DN) hydrogels.^[^ ^34^ ^]^ It is observed that the HSNMs proposed in this work provide a considerably larger *σ*_b_‐*ε*_b_ space than these typical soft materials. In specific, HSNMs offer a much larger stretchability (up to ∼450%) than previous SNMs, and provide higher tensile strengths than all of the other representative soft materials considered here. These results illustrate that the proposed HSNM design overcomes the classical conflict between high stretchability and high strength to a certain extent, i.e., have the ability to improve stretchability without loss of tensile strength (access to the right top region of Ashby plot). Moreover, distinct from the solid soft materials fabricated completely by chemical synthesis, the proposed HSNMs are capable of precisely achieving any stretchability and strength lower than the maximum value (orange region, Figure 1h) by rationally tailoring the geometric constructions (i.e., key parameters and topologies) and selecting appropriate constituent materials. Specifically, three totally different class of constituent materials are considered in this work, including polymer, metal, and polymethylmethacrylate (PMMA), to be elaborated later in this paper.

### 2.2 Stretchability and Strength of 3D Printed HSNMs Under Uniaxial Loading

**Figure 2:**
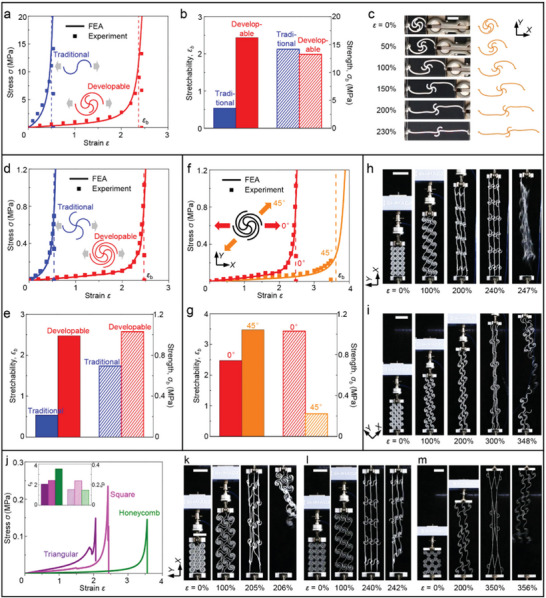
Experimental and FEA results of mechanical properties and deformed configurations for HSNMs fabricated by 3D printing. a) Stress–strain curves for building‐block specimens with developable nodes and traditional point‐shaped nodes. Insets show geometric configurations of the building‐block structures, and the dashed lines denote the fracture strain (i.e., stretchability, *ε*_b_) predicted by FEA. b) Measured stretchability (*ε*_b_) and strength (*σ*_b_) of the building‐block specimens applied in (a). c) Optical images and corresponding FEA results for the building‐block specimens with developable nodes under different levels of applied strains. d) Stress–strain curves for a HSNM with developable nodes and a SNM with traditional nodes. Insets show the geometric configurations of the unit‐cell structures, and the dashed lines denote the stretchability (*ε*_b_) predicted by FEA. e) Measured stretchability (*ε*_b_) and strength (*σ*_b_) of the HSNM and the SNM applied in (d). f) Stress–strain curves for a HSNM along two typical stretching directions (0° and 45° relative to *X*‐axis). g) Measured stretchability (*ε*_b_) and strength (*σ*_b_) for the HSNM along two typical directions. h,i) Optical images of deformation sequences for the HSNM specimen under stretching along 0°(h) and 45°(i), respectively. j) Measured stress–strain curves for HSNMs with different lattice topologies (triangular, square, and honeycomb). Inset shows the stretchability (*ε*_b_) and strength (*σ*_b_) of these HSNMs. k–m) Deformation sequences of the HSNMs with triangular (k), square (l), and honeycomb (m) topologies, respectively. Scale bars, 20 mm in (c), and 100 mm in (h), (i), as well as (k–m).

**Figure** **2**a provides experimental and finite element analyses (FEA) results of the nonlinear stress–strain curves for typical building‐block structures of proposed HSNMs and traditional SNMs. The experimental specimens are made of hard polymer (VeroPureWhite, Stratasys) and fabricated by a 3D printer (Object 350, Stratasys), with thickness *t* = 3 mm. The key geometric parameters of the building‐block specimens with developable node are set to $\bar{r}$ = 0.3, *φ* = 180°, *θ* = 180°, and $\bar{w}$ = 0.04, respectively. Note that the normalized width ($\bar{w}$) and the arc angle (*φ*_0_) of horseshoe microstructures are fixed as $\bar{w}$ = 0.04 and *φ*_0_ = 180° for both of the specimens with developable node and traditional node, which ensure a fair comparison for them. To provide a full picture of the proposed developable node, a complete structure of the developable node (four arcs for square HSNMs, including two suspended arcs) is reserved in the building‐block specimen, as the insets shown in Figure 2a. Although the developable nodes possess more complex constructions than the traditional point‐shaped nodes, the preparation process of HSNMs with developable nodes is not much more complicated than that of SNMs with point‐shaped nodes, since both of them are applicable for many well‐established manufacture techniques (e.g., 3D printing, photolithography, laser cutting, and precision casting). The dashed lines in Figure 2a denote the stretchabilities (*ε*_b_) predicted by FEA, based on the measured material parameters of constituent polymer (elastic modulus *E*_s_ = ∼1000 MPa, and elongation at break *δ* = ∼15%). It can be observed that both of the J‐shaped stress–strain (*σ–ε*) responses and the stretchabilities (*ε*_b_) predicted by FEA agree well with the experimental measurements. Figure 2b presents the comparison of stretchability (*ε*_b_) and tensile strength (*σ*_b_) between the building‐block specimens with developable node and traditional node. An obvious improvement of stretchability (more than 400% in relative) can be observed by applying the proposed developable node into building‐block structures, while the strength exhibits a negligible reduction (∼5% in relative). The improvement of stretchability can be mainly attributed to the additional deformation in terms of unfolding and bending offered by the developable node (Figure 2c). As shown in Figure 2c, the deformation of the building‐block specimen with developable node starts with the unbending of horseshoe microstructure, followed by the unfolding of developable node, and finally the unbending of node arcs. The FEA results (right) give accurate predictions on the deformed configurations which agree very well with the experimental photos (left) during the whole stretching process.

Figure 2d–m presents the experimental and FEA results of mechanical properties for HSNMs proposed in this work. These network specimens are made of the same constituent material (3D printed polymer) as the building‐block specimens in Figure 2a–c. The key geometric parameters of the network specimens in Figure 2d–i are identical to that of the building‐block specimens ($\bar{r}$ = 0.3, *φ* = 180°, *θ* = 180°, and $\bar{w}$ = 0.04), while the normalized width of the network specimens in Figure 2j–m are set to $\bar{w}$ = 0.02. Figure 2d,e shows a comparison of stress–strain (*σ–ε*) response, stretchability (*ε*_b_), and tensile strength (*σ*_b_) between the proposed HSNMs and the traditional SNMs. Here, to compare these mechanical properties in a fair manner and provide an intuitive illustration for the differences of stretchability, the geometric configurations of the horseshoe microstructures (i.e., normalized width $\bar{w}$ and arc angle *φ*_0_) and the lattice topology of the network structures for the traditional SNM specimen are set to be consistent with those of HSNM specimen. Similar to the results of building‐block structures (Figure 2a,b), the stretchability (∼250%) of HSNMs with developable nodes is much larger than that (∼50%) of SNMs with traditional nodes. Moreover, the HSNM specimen even provides a higher tensile strength than the SNM specimen, as shown in Figure 2e. It should be noted that in this situation of comparison (i.e., the normalized width is fixed as $\bar{w}$ = 0.04), the relative density ($\bar{\rho }$) of HSNM specimen is higher than traditional SNM specimen. Since the relative density also represents an important parameter of porous materials, we further compare the mechanical properties of proposed HSNMs and traditional SNMs with a same relative density ($\bar{\rho }$ = ∼0.28) to clarify the probable influence of the relative density, as shown in Figure S4 (Supporting Information). It can be observed that the improvement of stretchability (from ∼32% to ∼250%) for HSNM specimen is even more notable in comparison to traditional SNM specimen, while the tensile strength shows a slight variation. These results indicate that the improvement of stretchability is mainly attributed to the additional deformability provided by the proposed developable node design, rather than the increasement of relative density.

Figure 2f–i elucidates the anisotropy of mechanical properties for HSNMs with square topology. For a HSNM with fixed geometric construction, the uniaxial stress–strain responses along two typical loading directions (0° and 45° relative to *X*‐axis) are clearly different from each other, as shown in Figure 2f. Specifically, the stretchability of HSNMs under the uniaxial stretching along 45° is larger than that along 0°, while the tensile strength substantially reduces along 45° (Figure 2g). Results of the mechanical properties (i.e., stress–strain curve, stretchability, and strength) from experiments and FEA agree well for all of these specimens shown in Figure 2d–g. Figure 2h,i presents the optical images during the whole tensile process of HSNM specimens along the loading direction of 0° and 45°, respectively. The experimental photos of deformation sequences for the SNMs with traditional nodes are provided in Figure S5 (Supporting Information). Take the 3D printed HSNM specimen applied in Figure 2d–i as an example, Figure S6 (Supporting Information) further provides the stress–strain curves of HSNMs under cyclic loading, which indicates the stability and the recoverability of the mechanical properties. Note that the cyclic stress–strain curve falls more slightly as loading cycle increased, and both of the residual stretch and the hysteresis in the first cycle are much larger than the subsequent cycles. Moreover, Figure 2j presents the experimental results of stress–strain (*σ–ε*) curves for HSNM specimens with three different topologies (i.e., triangular, square, and honeycomb), and the comparison of stretchabilities (*ε*_b_) and strengths (*σ*_b_) between these three specimens is shown in the inset of Figure 2j. Both of the stretchability and strength of triangular HSNM specimen are lower than those of square HSNM specimen. Although the honeycomb HSNM specimen provide the highest stretchability among these three specimens, its strength is obviously lower than the strength of square HSNM specimen. Figure 2k–m provides the optical images of deformation sequences for the HSNMs with triangular, square, and honeycomb topologies, respectively, which further indicates the differences of stretchability and deformation mode for these HSNMs. Note that the HSNMs with square topology enable the combination of large stretchability and high strength, thus this work mostly focuses on the mechanical responses of square HSNMs, expect for the special discussions on the effect of lattice topology.

### 2.3 Advantages of the HSNMs with Developable Node Design Over Other SNMs

**Figure 3:**
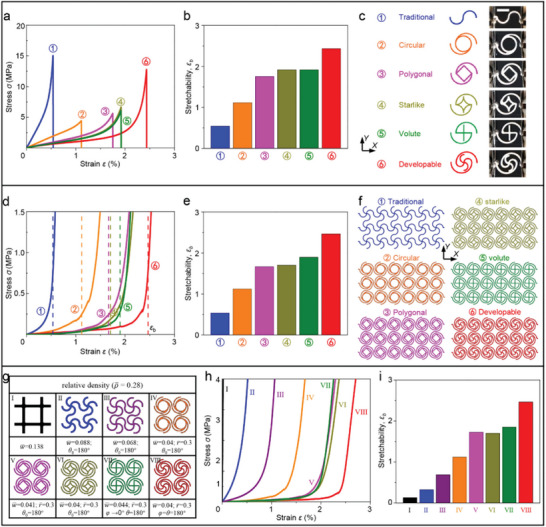
Stress–strain curves and stretchabilities for SNMs with various types of lattice nodes. a) Experimental results of stress–strain curves for building‐block structures of 3D printed SNMs with different types of nodes (e.g., traditional point‐shaped nodes, circular nodes, polygonal nodes, starlike nodes, volute nodes, and developable nodes). b) Measured stretchability (*ε*_b_) of these building‐block specimens. c) Schematic illustrations and optical images of the building‐block structures studied in (a) and (b). d) FEA results of stress–strain curves for SNMs with various types of lattice nodes, based on the measured mechanical properties of 3D printed constituent materials. The dashed lines denote the stretchability calculated by FEA. e) Comparison of the predicted stretchability (*ε*_b_) for these SNMs. f) Geometric configurations of the SNM specimens adopted in (d) and (e). g) Schematic illustrations (2 × 2 unit cells) and geometric parameters of traditional lattice materials (I), traditional SNMs with point‐shaped nodes (II and III), SNMs with different lattice nodes (IV–VII), and HSNMs with developable nodes (VIII), while the relative density of these network specimens are fixed as $\bar{\rho }$ = ∼0.28. h) FEA results of stress–strain curves for network specimens shown in (g). i) Comparison of the predicted stretchability (*ε*_b_) for these network specimens. Scale bars, 15 mm in (c).

Most of the existing SNMs are designed from the perspective of curved microstructures which used to connect the lattice nodes, while the lattice nodes actually offering another important route for the design of SNMs. Apart from the developable nodes studied above, three other types of nodes (i.e., polygonal node, starlike node, and volute node) are proposed in this section for constructing a systemic investigation of node‐based design strategy, as shown in **Figure** **3**. The polygonal node is formed by connecting the adjacent ends of microstructures with straight lines, and the starlike node is constructed by arcs in a similar manner, while the volute node is formed by connecting the opposite ends of microstructures with straight lines. It is noteworthy that the volute node can be obtained by setting the node angle (*φ*) of developable node tends to zero (i.e., *φ* → 0). The circular node shown in Figure 3 is proposed by Liu et al. in 2021,^[^ ^55^ ^]^ but it is only used to make up triangular SNMs in that previous work. Distinct from the traditional (point‐shaped) node and circular node which possess the identical geometry for SNMs with different lattice topologies, geometry of the new node designs proposed in this paper (i.e., ③–⑥ in Figure 3) depends highly on the topologies, as shown in Figure 1f and Figure S7 (Supporting Information). Specifically, the vertex number of polygonal node and starlike node, as well as the arc number of developable node and volute node, are consistent with the number of microstructures connected to the lattice node.

Figure 3a,b presents the experimental results of stress–strain (*σ–ε*) curves and stretchabilities (*ε*_b_) for the building‐block structures with various lattice nodes. Schematic diagrams of these building‐block structures and the corresponding 3D printed specimens are shown in Figure 3c. Note that the complete configurations of these lattice nodes are reserved in the building‐block structures of square SNMs. To ensure a fair comparison, the key geometric parameters (e.g., the normalized width and arc angle of the microstructures, as well as the size of these untraditional nodes) of these building‐block structures are fixed as the same value. The building‐block specimen with developable node offers higher stretchability than the other specimens. The stretchabilities of specimens with polygonal node, starlike node and volute node are similar to each other, and all of them are higher than the stretchability of specimens with circular node, which can be attributed to the reduction of self‐overlay region (the effect of self‐overlay will be elaborated later). The specimen with traditional node possesses the lowest stretchability, but offers a much higher tensile strength than the specimens with other types of nodes besides developable node. The above results further indicate the abilities of developable node design to offer fairly high stretchability without loss of strength. Figure 3d–f provides the similar results for network specimens consisting of the building‐block structures in Figure 3a–c. Here, the stress–strain (*σ–ε*) curves and stretchabilities (*ε*_b_) are predicted by FEA method, and the geometric constructions of FEA models are shown in Figure 3f, which contain the same scale of unit cells as the experimental specimens used in Figure 2h. It is observed that the distribution of mechanical properties for network specimens with different nodes shows good consistency with that of building‐block specimens. The HSNMs with developable nodes offers higher stretchability than all of the other SNMs investigated here. The results in Figure 3d–f are obtained by fixing the geometry of microstructures and the size of lattice nodes for different network specimens, thus the relative density ($\bar{\rho }$) of these network specimens differ from each other. To clarify the universal superiority of the proposed developable nodes, Figure 3g–i provides the comparison of different class of network specimens by fixing the relative density as $\bar{\rho }$ = ∼0.28. The geometric configurations and the corresponding key parameters of these network specimens are presented in Figure 3g, note that the traditional lattice material (I) and another traditional SNM (III) are considered as additional cases. It is interesting to observe that the HSNMs with developable nodes (VIII) and the SNMs with circular nodes (IV) possess a same relative density ($\bar{\rho }$) for adopting the same geometric parameters. Figure 3h,i shows the FEA results of stress–strain (*σ*–*ε*) curves and stretchabilities (*ε*_b_) for the network specimens in Figure 3g, respectively. Similar to the results shown in Figure 3d–f, the HSNMs with developable nodes are capable of offering the highest stretchability in comparison to all of the other network specimens.

**Figure 4:**
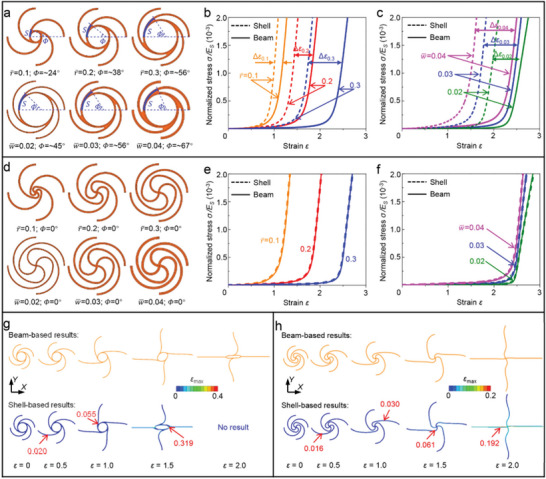
Illustration of the advantages for proposed developable nodes in contrast to existing circular nodes. a) Schematic illustration of the unit cells for circular‐node SNMs with various normalized radius ($\bar{r}$ = 0.1, 0.2, and 0.3) and normalized width ($\bar{w}$ = 0.02, 0.03, and 0.04). The self‐overlap segments around the nodes are marked in blue. b) Shell‐based FEA (model with the same geometric configurations as the experimental specimens, with self‐overlap) and beam‐based FEA (model constructed with wires based on theoretical design, without self‐overlap) results of normalized stress–strain curves for circular‐node SNMs with varying $\bar{r}$. The other key geometric parameters are fixed as *θ* = 180° and $\bar{w}$ = 0.03. Δ*ε*_0.1_, Δ*ε*_0.2_, and Δ*ε*_0.3_ denote shifts of the stress–strain curves for SNMs with $\bar{r}$ = 0.1, 0.2, and 0.3, respectively. c) Similar results for circular‐node SNMs with different $\bar{w}$. The other key geometric parameters are fixed as *θ* = 180° and $\bar{r}$ = 0.3. d) Schematic illustration of the unit cells for HSNMs with various $\bar{r}$ (0.1, 0.2, and 0.3) and $\bar{w}$ (0.02, 0.03, and 0.04). e) Shell‐based FEA and beam‐based FEA results of normalized stress–strain curves for HSNMs with varying $\bar{r}$. The other key geometric parameters are fixed as *φ* = 180°, *θ* = 180°, and $\bar{w}$ = 0.03. f) Similar results for HSNMs with different $\bar{w}$. The other key geometric parameters are fixed as *φ* = 180°, *θ* = 180°, and $\bar{r}$ = 0.3. g) Shell‐based FEA and beam‐based FEA results of deformed configurations for a representative unit cell of circular‐node SNMs under different level of applied strain. Distribution of the maximum principal strain (*ε*_max_) in the unit‐cell structures are provided by shell‐based FEA, and the locations and the values of peak *ε*_max_ are marked in these images. The key geometric parameters are fixed as *θ* = 180°, $\bar{r}$ = 0.2, and $\bar{w}$ = 0.03. h) Similar results for a representative unit cell of HSNMs with developable nodes. The key geometric parameters are fixed as *φ* = 180°, *θ* = 180°, $\bar{r}$ = 0.2, and $\bar{w}$ = 0.03.

The improvement of stretchability for the developable node design can be attributed to three aspects: (1) additional deformation in terms of unfolding and bending; (2) avoidance of self‐overlay; and (3) reduction of stress/strain concentration. Note that the first aspect has been clarified in Section 2.1 and 2.2. To expound the other two aspects for the advantages of HSNMs with developable nodes, the SNMs with circular nodes are used as an example for comparison, as shown in **Figure** **4**. Note that the results presented in Figure 4 are based on a linear elastic constituent material. For SNMs with circular nodes, there always exist self‐overlays around the nodes in fabricated specimens (Figure 4a). The degree of the self‐overlays can be described by the arc length (*S*) or the arc angle (*Φ*) of overlapped regions, which is related to the geometric constructions of the nodes, as shown in Figure 4a. It is observed that the self‐overlay around the nodes become more and more serious (i.e., a larger *Φ*) with increasing the normalized radius ($\bar{r}$) and the normalized width ($\bar{w}$). Nevertheless, the theoretical design of SNMs usually takes no account of the self‐overlay factor (since the regardless of width in geometric modeling), thereby resulting in a significant difference in the stretchability between the initial design and the practical fabrication. The as‐designed and as‐fabricated specimens can be precisely modeled by FEA based on beam elements and shell elements, respectively. Figure 4b presents the beam‐based FEA and shell‐based FEA results of normalized stress–strain curves for circular‐node SNMs with different normalized radius ($\bar{r}$ = 0.1, 0.2, and 0.3). For all of these three SNMs, the stress–strain curves move leftward from the beam‐based result to the shell‐based result, which indicate the reduction of stretchability. Moreover, the leftward shifts of stress–strain curves become more evident as $\bar{r}$ increases, i.e., Δ*ε*_0.1_ < Δ*ε*_0.2_ < Δ*ε*_0.3_. Similarly, a more obvious leftward movement of stress–strain curves can be observed with increasing $\bar{w}$, as shown in Figure 4c. The above results indicate that the self‐overlay of SNMs with circular nodes is inevitable in practical applications, which sharply reduces the stretchability. Moreover, the degree of self‐overlay is highly related to the geometric constructions of SNMs, which makes it very difficult to be controlled in fabrications. Figure 4d presents various unit cells of proposed HSNMs with different geometric parameters, and it is interesting to note that there's no self‐overlay for all of the HSNMs with developable nodes. Figure 4e,f provides the beam‐based FEA and shell‐based FEA results of normalized stress–strain curves for HSNMs with different normalized radius ($\bar{r}$) and normalized width ($\bar{w}$), respectively. Results from beam‐based FEA and shell‐based FEA agree very well for all of the HSNMs, which further provides quantitative evidence for the inexistence of self‐overlay.

Figure 4g presents the comparison of deformed configurations between beam‐based FEA results and shell‐based FEA results for a typical unit cell of circular‐node SNMs. It is observed that the deformed configurations obtained by beam‐based FEA are close to that of the shell‐based FEA at small applied strains (e.g., *ε* < 1.0). However, significant differences come into sight at relatively large applied strains (e.g., *ε* > 1.0), as shown in Figure 4g. Apart from the deformed configurations, the distributions of maximum principal strains (*ε*_max_) in the beam‐based model and the shell‐based model show clear distinctions, as shown in Figure S8 (Supporting Information). As for the HSNMs with developable nodes proposed in this work, both of the deformed configurations and the *ε*_max_ distributions obtained by beam‐based FEA and shell‐based FEA are in good accordance, as shown in Figure 4h and Figure S9 (Supporting Information). The shell‐based FEA results of locations and values of the peak *ε*_max_ for unit cells with circular node and developable node are marked in Figure 4g,h. For the SNMs with circular nodes, an evident stress (strain) concentration happens at the corner of node and microstructures, as shown in Figure 4g. It is clear that the effect of stress concentration is reduced by adopting a developable node design (Figure 4h). For example, the spatial maximum of *ε*_max_ in unit cell with developable node is ∼0.061, much smaller than that (∼0.319) in unit cell with circular node, in the case of the same applied strain (*ε* = 1.5). So far, all of the three aspects of advantages for the developable node design have been clarified. In addition, the SNM designs with polygonal node, starlike node, and volute node can also reduce the degree of self‐overlay to some extent. For instance, we have established a theoretical model for predicting the stress–strain curves of triangular SNMs with solid circular nodes.^[^ ^55^ ^]^ This model failed to describe the experimental specimens with self‐overlay (Figure S10a,c, Supporting Information), unless adopting a modification factor (*K*) with complicated form. Nevertheless, for the SNMs with solid starlike nodes, the theoretical model can be well modified to predict the stress–strain curves for both of the beam‐based FEA model and shell‐based FEA model (with the same geometric construction as experimental specimen), as shown in Figure S10b,d (Supporting Information). More importantly, the avoidance of self‐overlay not only ensure a high stretchability of HSNMs in practical fabrication, but also make it possible to precisely predict the mechanical properties of HSNMs by beam‐based FEA method, which is able to efficiently reduce the computational efforts of simulations.

### 2.4 Effect of the Key Geometric Parameters and the Lattice Topologies of HSNMs

**Figure 5:**
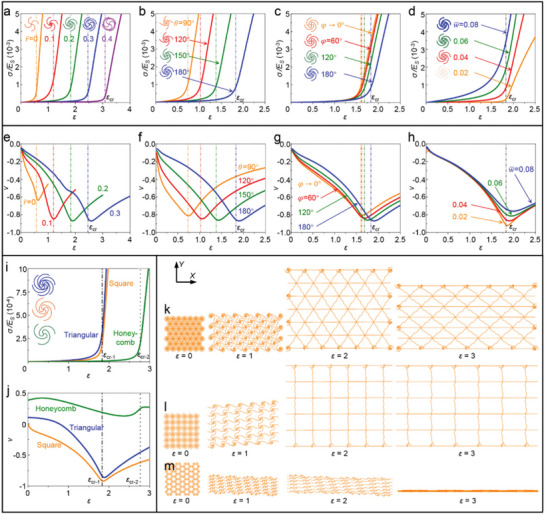
Effects of key geometric parameters and lattice topologies on the mechanical properties for HSNMs with developable nodes. a–d) Effects of normalized radius ($\bar{r}$) (a), joint angle (*θ*) (b), node angle (*φ*) (c), and normalized width ($\bar{w}$) (d) on the normalized stress‐strain curves for HSNMs. e–h) Effect of $\bar{r}$ (e), *θ* (f), *φ* (g), and $\bar{w}$ (h) on the nonlinear Poisson's ratios for HSNMs. The chain‐dotted lines in (a–h) denote the critical strain (*ε*_cr_) of the square HSNMs. i,j) Effects of lattice topologies on the normalized stress–strain responses (i) and nonlinear Poisson's ratios (j) for the HSNMs with developable nodes. The chain‐dotted lines denote the critical strain (*ε*_cr‐1_) of HSNMs with triangular and square topologies, and the dotted lines denote the critical strain (*ε*_cr‐2_) of honeycomb HSNMs. k–m) Deformed configurations of the HSNMs with triangular (k), square (l), and honeycomb (m) topologies, at different levels of applied strains. All of the HSNMs under uniaxial stretching along *X*‐axis.

**Figure** **5** systematically elucidates the effect of geometric constructions on the stress–strain (*σ–ε*) curves and the Poisson's ratios (*ν*) of HSNMs with developable nodes under uniaxial stretching. Here, plentiful FEA models have to be established for parametric analysis. Note that the shell‐based FEA models are very time‐consuming due to a huge number of elements, the FEA techniques applied in this section are based on the beam elements, whose accuracy for HSNMs has been verified in Figure 4. The schematic diagrams of unit cells of HSNMs corresponding to the stress–strain curves are provided as insets. In addition, the critical strain (*ε*_cr_, the applied strain to fully align the building‐block structures of HSNMs along stretching direction) of these HSNMs are marked as dash‐dotted lines (dotted lines for honeycomb HSNMs), which can be given by,

(4) εcr−1=2φ0r¯0+φr¯sinφ/2−1εcr−1=fortriangularandsquarelatticetopologies,εcr−2=83φ0r¯0+4φr¯3sinφ/2−1εcr−2=forhoneycomblatticetopologies.

Note that the critical strain acts as an important parameter to describe the tendency of stress–strain curves, i.e., the stress–strain curves almost raise linearly when the applied strain larger than the critical strain (*ε > ε*_cr_), thus the *ε*_cr_ is directly related to the stretchability (*ε*_b_). Figure 5a presents the normalized stress–strain curves for square HSNMs with different normalized radius ($\bar{r}$), by fixing the node angle (*φ =* 180°), the joint angle (*θ =* 180°), and the normalized width ($\bar{w}$ = 0.04). The J‐shaped stress–strain curve shifts rightward equably and prominently as the $\bar{r}$ increases, indicating an effective improvement of stretchability. In specific, the critical strain of HSNMs increases linearly from *ε*_cr_ = ∼0.57 to *ε*_cr_ = ∼3.08 with increasing the normalized radius from $\bar{r}$ = 0 to $\bar{r}$ = 0.4 (Figure S11a, Supporting Information). Figure 5b and Figure S11b (Supporting Information) present the similar results for different joint angles (*θ*), with fixed normalized radius ($\bar{r}$ = 0.2), node angle (*φ =* 180°) and normalized width ($\bar{w}$ = 0.04). Both of the stress–strain curves and critical strains can be tuned in a wide range by adjusting the *θ*. The effect of node angle (*φ*) on the normalized stress–strain curve is illustrated in Figure 5c, with fixed normalized radius ($\bar{r}$ = 0.2), joint angle (*θ =* 180°) and normalized width ($\bar{w}$ = 0.04). It is observed that the node angle (*φ*) possesses less ability than the normalized radius ($\bar{r}$) and the joint angle (*θ*) to regulate the stress–strain curves, due to the little effect of node angle on the critical strain (Figure S11c, Supporting Information). Note that the stress–strain curve shifts more evidently (the *ε*_cr_ increases more rapidly) as the node angle approaches to 180°. Moreover, the arcs of horseshoe microstructures are going to be tangent to the arcs of developable nodes as *φ =* 180°, thus the stress concentration can be sufficiently reduced, thereby offers a higher stretchability. The normalized curvatures ($\bar{K}$) of the circular arcs of developable nodes are related to both of the normalized radius ($\bar{r}$) and the node angle (*φ*), which can be given as,

(5) $$\bar{K}=2\sin\left(\varphi /2\right)/\bar{r}$$

It is interesting to note that different sets of normalized radius ($\bar{r}$) and node angle (*φ*) can result in a same value of curvature ($\bar{K}$) but entirely different mechanical properties. For example, the normalized curvature ($\bar{K}$) equals to 5 for both developable nodes with “$\bar{r}$ = 0.2, *φ* = 60°” and “$\bar{r}$ = 0.4, *φ* = 180°”, however, leading to distinct stress–strain responses (Figure S12, Supporting Information). Figure 5d presents the normalized stress–strain curves for four normalized widths ($\bar{w}$), by fixing the normalized radius ($\bar{r}$ = 0.2), the node angle (*φ =* 180°), and the joint angle (*θ =* 180°). The stress–strain curve shifts upward gradually as the normalized widths ($\bar{w}$) increases, indicating an improvement of the tensile strength (*σ*_b_) of HSNMs. Nevertheless, the $\bar{w}$ has no effect on the *ε*_cr_ (Figure S11d, Supporting Information), thus shows a negligible influence on the stretchability (*ε*_b_) of HSNMs. It is noteworthy that the accessible range of $\bar{w}\;$is related to the other geometric parameters, in order to avoid the potential self‐overlay between two adjacent building‐block structures. For example, for a representative HSNM with *φ* = *θ* = 180°, the normalized width ($\bar{w}$) and normalized radius ($\bar{r}$) should satisfy the following constraint equations,

(6) $$\bar{w}<0.25-\sqrt{{\left(0.25-0.5\bar{r}\right)}^{2}+{\left(0.5\bar{r}\right)}^{2}}$$

It is interesting to note that the maximum value of $\bar{w}$ is not monotonically decreasing as $\bar{r}$ increases, which further indicates the feasibility of a combination of high stretchability and high tensile strength.

Figure 5e–h shows a full picture of the effect of these key geometric parameters on the nonlinear Poisson's ratios (*ν*) of square HSNMs. The square HSNMs with developable nodes are able to offer a wide range of negative Poisson's ratios (e.g., from 0 to ‐0.9) by tuning the applied strains, and this accessible range of negative Poisson's ratios is larger than that (e.g., from 0 to ‐0.6) of traditional square SNMs ($\bar{r}$ = 0). The magnitude of negative Poisson's ratios come up to its maximum value as the applied strain gets close to the critical strain (i.e., *ε = ≈ε*_cr_), thus the response of nonlinear Poisson's ratio under large deformation can be also well regulated by the normalized radius ($\bar{r}$), the node angle (*φ*), and the joint angle (*θ*) (Figure 5e–g). The normalized width ($\bar{w}$) has negligible effect on the curves of Poisson's ratios, except for the peak value around *ε*_cr_ (Figure 5h). It is also noteworthy that the initial linear Poisson's ratios (i.e., the *ν* at *ε* = ∼0) are almost impervious to these geometric parameters, which approach to zero for all of the HSNMs investigated here.

Figure 5i–m illustrates the effect of lattice topology on the mechanical properties of HSNMs. The key geometric parameters for all of these three HSNMs with different topologies (i.e., triangular, square, honeycomb) are fixed as $\bar{r}$ = 0.2, *φ =* 180°, *θ =* 180°, and $\bar{w}$ = 0.02. In specific, Figure 5i presents the normalized stress–strain curves for these three lattice topologies, and the schematics of unit cells are provided as insets. The J‐shaped stress–strain curve of triangular HSNM closes to but higher than that of square HSNM for various applied strains, and both of these two curves increase rapidly after *ε*_cr‐1_ (*ε*_cr‐1_ denotes the critical strain of triangular and square HSNMs). Note that the critical strain (*ε*_cr‐2_) of honeycomb HSNM is much higher than *ε*_cr‐1_, thus the stress–strain curve of honeycomb HSNM is located in the rightmost among these curves. The nonlinear Poisson's ratio (*ν*) of HSNM with honeycomb topology is also distinct from that of HSNMs with triangular and square topologies, as shown in Figure 5j. Both of the triangular and square HSNMs provide access to a wide range of negative Poisson's ratios (e.g., from 0 to −1), while the honeycomb HSNM possess the positive Poisson's ratios. Note that the critical strains (*ε*_cr‐1_ and *ε*_cr‐2_) can be also used to describe the tuning point of the curves of Poisson's ratios. The effects of lattice topology on the mechanical properties of SNMs with circular nodes and volute nodes are provided in Figure S13 (Supporting Information), which demonstrate similar results with HSNMs. Figure 5k–m presents the FEA results of deformation sequences for HSNMs with triangular, square, and honeycomb topologies under uniaxial stretching along *X*‐axis, respectively. In accordance with the results shown in Figure 5j, the triangular and square HSNMs possess negative Poisson's ratios (the HSNMs expand along *Y*‐axis), and the magnitude of *ν* for square HSNM is slightly larger than triangular HSNM. The honeycomb HSNM shrink along *Y*‐axis under uniaxial stretching along *X*‐axis, which represents a typical positive Poisson effect. In addition, a kind of shear deformation occurs clearly in square and honeycomb HSNMs at relatively small applied strains (Figure 5l,m), which is mainly attributed to the less number of microstructures connected to the developable nodes and the antisymmetric geometry of building‐block structures.

### 2.5 Demonstration of HSNMs Made of Metallic Constituent Material

**Figure 6:**
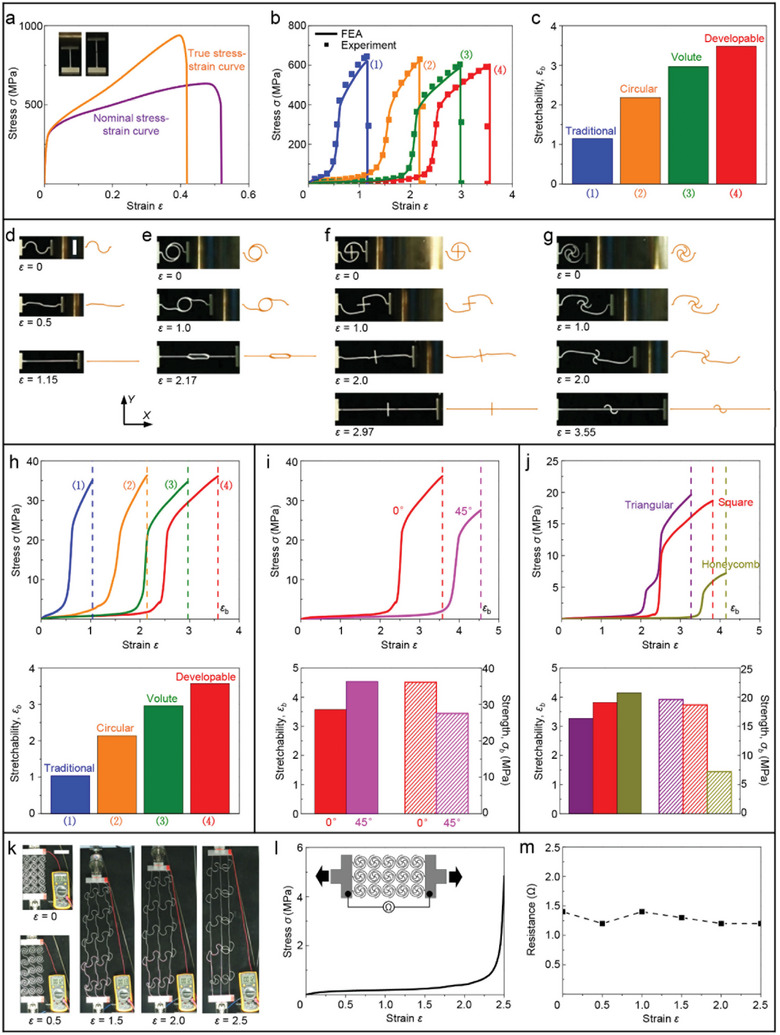
Experimental and FEA results of mechanical properties and electrical performances for HSNMs made of metal. a) Measured stress–strain curves of the stainless steel, which used as the constituent material for HSNM specimens. Insets show the optical images of undeformed (left) and fractured (right) test specimen. b) Stress–strain curves for building‐block specimens with traditional nodes, circular nodes, volute nodes, as well as developable nodes. c) Measured stretchability (*ε*_b_) of the building block specimens related to (b). d–g) Optical images and corresponding FEA results of deformed configurations for the building‐block specimens with traditional nodes (d), circular nodes (e), volute nodes (f), and developable nodes (g), at different levels of applied strains. h) FEA results of stress–strain curves (top) and predicted stretchability (*ε*_b_) (bottom) for the SNM specimens with traditional nodes, circular nodes, and volute nodes, as well as HSNM specimen with developable nodes. i) FEA results of stress–strain curves (top) and predicted ultimate mechanical properties (bottom) for a HSNM specimen with developable nodes stretching along two representative directions (0° and 45° relative to *X*‐axis). j) FEA results of stress–strain curves (top) and predicted ultimate mechanical properties (bottom) for HSNMs with three typical lattice topologies (triangular, square, and honeycomb). k) Photographs of deformed configurations and electrical resistances for a HSNM specimen at different tensile strains. l) Experimental results of stress–strain curves for this HSNM specimen. The inset shows geometric configuration (the key parameters are fixed as $\bar{r}$ = 0.3, *φ* = 180°, *θ* = 180°, and $\bar{w}$ = 0.02) and measurement circuit of the HSNM specimen. m) Measured resistances versus strains corresponding to (l). Scale bars, 15 mm in (d)–(g), and 80 mm in (k).

Another attractive feature of the HSNMs proposed in this work is that they can be fabricated in a variety of constituent materials, which is distinct from most of the chemosynthetic soft materials and provides one more pathway to tailor the mechanical properties. In this section, a type of metallic constituent materials is used to make up HSNMs, and the constructed HSNMs enable furtherly improvement of stretchability and strength. **Figure** **6**a presents the measured nominal stress–strain curve of stainless steels (SUS304) which acts as the constituent material of metallic HSNMs. The optical images of experimental specimens before loading and just broken are shown as insets in Figure 6a. The true stress–strain curve adopted in FEA can be obtained based on the nominal stress–strain curve by

(7) $$\sigma_{\mathrm{ture}}=\sigma_{\mathrm{nominal}}\left(1+\varepsilon_{\mathrm{nominal}}\right)\;\mathrm{and}\;\varepsilon_{\mathrm{ture}}=\ln\left(1+\varepsilon_{\mathrm{nominal}}\right)$$

Figure 6b provides experimental and FEA results of stress–strain curves for the building‐block structures of HSNMs and SNMs with three different nodes, including traditional point‐shaped node, circular node and volute node. The metallic building‐block specimens studied here have the same geometric parameters as the 3D printed specimens in Figure 3c. All of the nonlinear stress–strain curves, tensile strengths (the peak value of stress), and stretchabilities (the applied strain at fracture) predicted by FEA show good agreements with the experimental measurements. Note that the strengths of these four building‐block specimens are very close to the measured strength of stainless steels, since the curved microstructures are fully straightened when a fracture happens (Figure 6d–g). Specifically, the measured stretchabilities of these building‐block specimens are compared in Figure 6c. The building‐block specimen with developable node possesses a clearly higher stretchability than the other specimens, for example, the stretchability (∼350%) of developable node is more than three times higher than that (∼110%) of traditional node. Figure S14 (Supporting Information) further provides the experimental results of stress–strain curves for building‐block structures made of PMMA (a typical brittle constituent material), considering a variety of lattice nodes. Note that both of the metallic and PMMA‐based building‐block specimens with developable nodes show higher stretchability than the other kinds of nodes, which indicates the ability of developable node design to improve the stretchability is applicable for a wide range of constituent materials (e.g., elastic materials, ductile materials, and brittle materials). Figure 6d–g presents optical images and FEA results of the deformed configurations for metallic building‐block specimens with traditional node, circular node, volute node, and developable node, respectively. The deformations of different specimens can be captured accurately by FEA during the entire stretching process. Figure 6b,d–g fully demonstrates the validity of FEA to predict the mechanical properties of HSNMs made of metal.

Figure 6h presents the FEA results of nonlinear stress–strain responses for network specimens with traditional node, circular node, volute node, and developable node. The key geometric parameters of these network specimens are consistent with that of building‐block specimens studied above. The dashed lines denote the predicted stretchability (*ε*_b_) of these network specimens, and these *ε*_b_ are compared visually in the bottom panel of Figure 6h. The results of network specimens are consistent with that of building‐block specimens shown previously in Figure 6b,c, i.e., the HSNM specimen with developable nodes enables access to a much higher stretchability than other SNM specimens, without any loss of tensile strength. The anisotropic characteristic of metallic HSNM with square topology is illustrated in Figure 6i. Similar to the 3D printed HSNM specimens, the metallic HSNM specimen possesses a higher stretchability but a lower strength under the uniaxial stretching along 45°. Figure 6j presents the FEA results of stress–strain curves, stretchabilities, and strengths for metallic HSNM with triangular, square, and honeycomb topologies. Note that the effect of lattice topology on the mechanical properties of metallic HSNMs also has many similarities to the 3D printed HSNMs. Nevertheless, according to Figures 2 and 6, both of the stretchability and the strength of metallic HSNM specimens are much higher than that of 3D printed HSNM specimens. In addition, it is also noteworthy that the HSNMs made of conductive constituent materials have the potential to possess good electrical performance, which is attractive in the applications of stretchable electronics. To demonstrate the electrical performance of HSNMs, the resistance of a metallic HSNM specimen is measured at different levels of applied tensile strains, as shown in Figure 6k–m. Figure 6k provides the experimental results of deformed configurations and resistances of the HSNM specimen under uniaxial stretching, and the corresponding J‐shaped stress–strain curve is presented in Figure 6l. Figure 6m provides the variation of electrical resistance as applied strain increased, it is observed that the resistance of metallic HSNM specimens is capable of remaining steady throughout the whole stretching process.

## 3 Conclusion

In conclusion, this paper introduces a hierarchical‐inspired design strategy for SNMs by introducing a class of developable nodes to replace the traditional point‐shaped lattice nodes, which could significantly improve the stretchability (up to ∼450%) of SNMs without any loss of strength (more than ∼30 MPa). The proposed HSNMs with developable nodes provide access to a fascinating capability that both of the stretchability and the tensile strength higher than the conventional soft materials (e.g., PDMS) widely used as the substrate and/or encapsulation layer in the stretchable inorganic electronics. Combined experimental and FEA studies of the mechanical properties for HSNMs with developable nodes are provided in this work, and the measured stress–strain curves, stretchabilities, strengths, as well as deformed configurations, can be accurately predicted by FEA. Compared to the traditional SNMs with point‐shaped nodes, the proposed HSNMs could offer a ∼5 times higher stretchability. The node design method of SNMs is systematically presented. Compared to the other node designs (e.g., circular, polygonal, starlike, and volute), the developable node always has ascendancy over them. The achievement of high stretchability for the developable node design can be attributed to additional unfolding deformation, avoidance of self‐overlay, and reduction of stress concentration. Geometric parameters, lattice topologies, as well as loading directions, are found to have different level of effects on the mechanical properties of proposed HSNMs with developable nodes. The FEA based on the beam elements is shown as a reliable and efficient way to predict the J‐shaped stress–strain curves and nonlinear Poisson's ratios of HSNMs with different key geometric parameters ($\bar{r}$, *φ*, *θ*, and $\bar{w}$). Moreover, the ability of developable node design to improve the stretchability is applicable for a wide range of constituent materials (e.g., elastic material, ductile material, and brittle material). The metallic HSNMs made of stainless steel are demonstrated to possess higher stretchability and strength, as well as stable electrical resistance, which suggests the capabilities of proposed HSNM design in achieving high mechanical performance required in a broad range of practical applications.

## 4 Experimental Section

### Fabrication and Mechanical Test of HSNMs

The building‐block specimens and network specimens made of hard polymer constituent material (Figures 1, 2, 3) were fabricated by a 3D printer (Object 350, Stratasys, USA). A series of 3D geometric descriptions of the specimens were created by commercial software (SOLIDWORKS) and then turned into .stl files for the 3D printer. In specific, the network specimens exploited for measurement consist of 6 × 3 unit cells (six unit cells along loading direction) and two solid plates fixed to both ends of the network region. The specimens were printed with a typical photopolymer (VeroPureWhite), and the support material (SUP 706) around the specimens were removed by low pressure water jet. The specimens made of metallic constituent material (Figure 6) were prepared using a stainless steel (SUS 304) board (≈3 mm in thickness) and patterned by infrared laser cutting technique. A series of 2D geometric models of the specimens were created by commercial software (AUTOCAD) and then turned into .dxf files for a laser cutting machine (HAN'S LASER, China). The fabricating process of specimens made of PMMA (Figure S14, Supporting Information) was similar to that of metallic specimens, note that the PMMA boards were patterned by another laser cutting machine (VLS 3.50, Universal, USA). Measurements of force‐displacement curves, maximum elongations, and peak forces were conducted using a universal testing machine (SHIMADZU AGX‐X, Japan). To ensure a quasistatic loading process and a constant applied strain rate, the loading rate was fixed as 10 mm min^−1^ for all of the building‐block specimens, and 60 mm min^−1^ for network specimens. Using a lighttight black cloth as the background, a high magnification digital camera (CANON, Japan) captured in situ the deformed configurations of the building‐block specimens and network specimens during the entire stretching process.

### Finite Element Analysis

2D FEA performed using the commercial software ABAQUS (SIMULIA) were used to predict the stress–strain curves, stretchabilities, strengths, and stress–strain distributions of proposed HSNMs under uniaxial stretching, with the geometric nonlinearity taken into account. Four‐node bilinear reduced integrated shell elements (CPS4R) were used to model the experimental specimens of HSNMs, with use of the same geometric constructions and boundary conditions as that in experiments (Figures 1, 2, 3, 6). Quadratic Timoshenko beam elements (B22) were used to reveal the effect of key geometric parameters and lattice topologies of HSNMs, with sufficiently large computational models to avoid the boundary effect, and the Poisson's ratios were calculated using the deformations of a typical unit cell at the central area of the model (Figure 5). The FEA results based on these two types of elements were compared using the computational models consisting of 6 × 6 unit cells, and the boundaries of these models were allowed to deform freely along *Y*‐axis while applying displacement component to the loading boundaries along *X*‐axis (Figure 4). For all of the FEA models, refined meshes were adopted to ensure the computational accuracy. According to the experimental observations, a linear elastic constitutive relationship (with a Young's modulus of *E*_S_ = ∼1000 MPa) could be used to model deformations of 3D printed HSNM specimens, and a simple fracture criterion which based on the peak value of maximum principal strain in the entire models (elongation at break, *δ* = ∼15%) could be applied to predict the stretchabilities. As for metallic HSNM specimens made of SUS 304, an isotropic elastic–plastic constitutive relationship was adopted in the FEA, where the stress–strain curves and material parameters were measured in experiments.

## Conflict of Interest

The authors declare no conflict of interest.

## Supporting information

Supporting Information

## Data Availability

The data that support the findings of this study are available from the corresponding author upon reasonable request.
