# Supplementary material for: Designing Hierarchical Soft Network Materials with Developable Lattice Nodes for High Stretchability
Source: Adv Sci (Weinh). 2023 Jan 25;10(8):2206099. doi: 10.1002/advs.202206099 (PMC10015852; doi:10.1002/advs.202206099)
Supplement: Supplementary file 1 — Supporting Information [file ADVS-10-2206099-s001.pdf]

Supporting Information

Designing hierarchical soft network materials with developable lattice nodes for high stretchability

*Jianxing Liu, Haoyu Guo, Haiyang Liu, and Tongqing Lu**

[*] Prof. Tongqing Lu, Corresponding-Author
State Key Lab for Strength and Vibration of Mechanical Structures, Soft Machines Lab, Department of Engineering Mechanics
Xi'an Jiaotong University
Xi'an 710049, China
E-mail: tongqinglu@mail.xjtu.edu.cn

Dr. Jianxing Liu, Mr. Haoyu Guo, Mr. Haiyang Liu
State Key Lab for Strength and Vibration of Mechanical Structures, Soft Machines Lab, Department of Engineering Mechanics
Xi'an Jiaotong University
Xi'an 710049, China

Keywords: hierarchical-inspired design; soft network material; lattice node; stretchability; constituent material

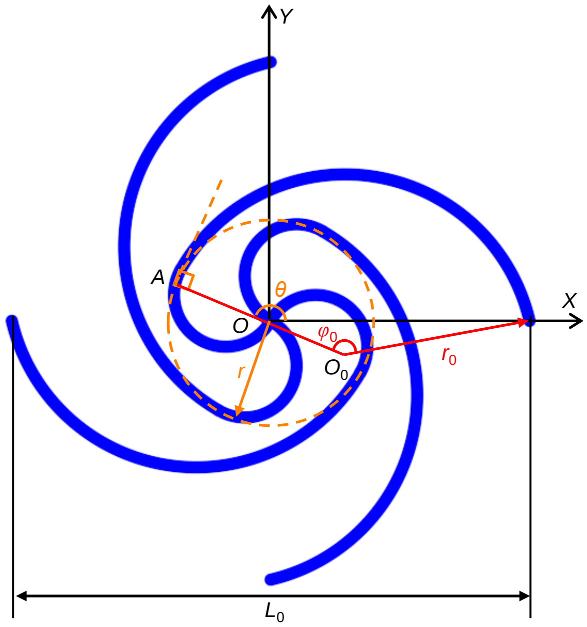

Figure S1. Schematic illustration of the unit-cell structure corresponding to Figure 1c, with marked geometric parameters of horseshoe-shaped microstructures, i.e., arc angle (φ_0) and radius (r_0).

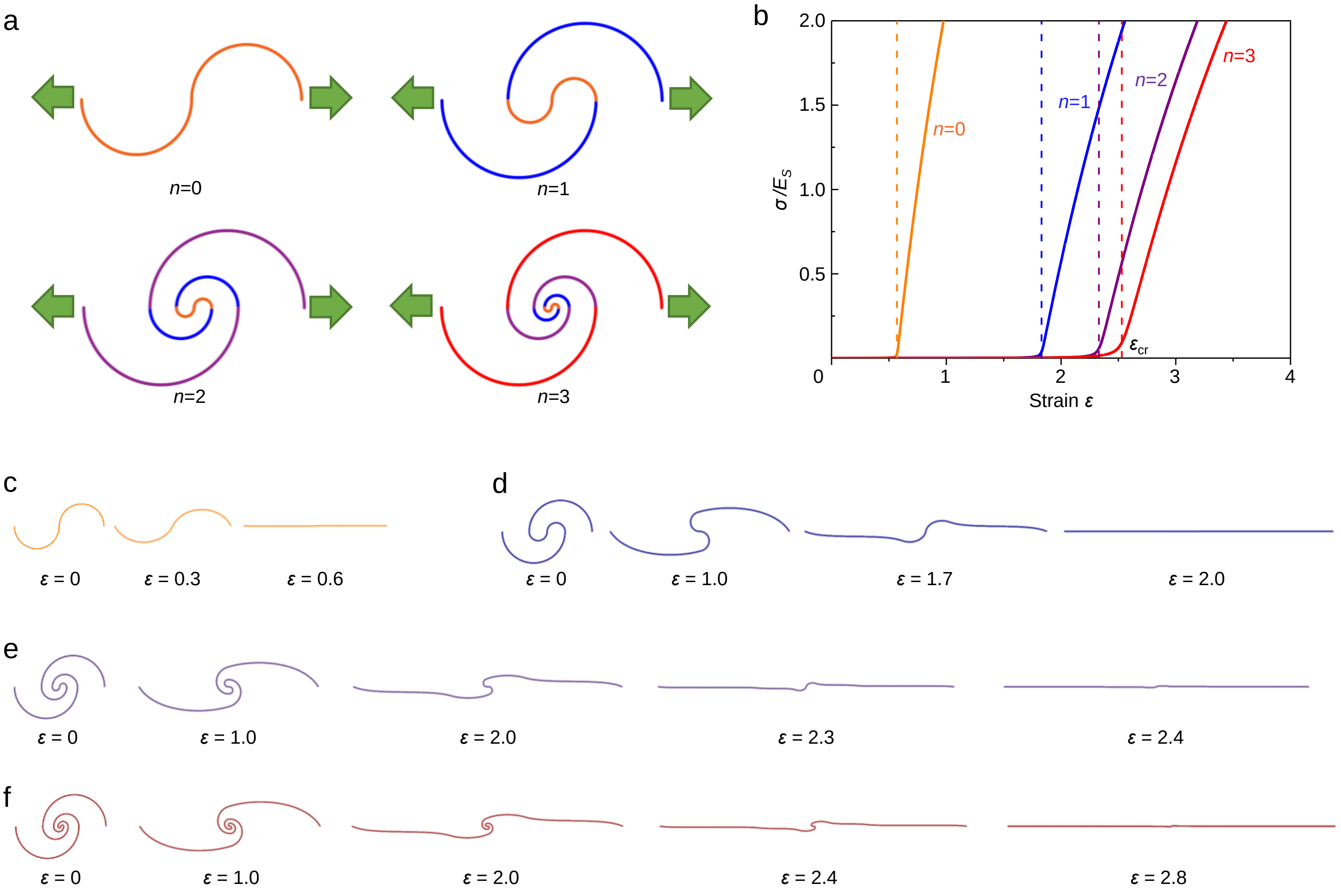

Figure S2. Mechanical properties and deformed configurations for building-block structures of HSNMs with different hierarchical orders (n). a) Schematic illustrations of building-block structures with hierarchical order n = 0, 1, 2, 3. b) Normalized stress-strain curves of these hierarchical building-block structures under uniaxial stretching. The dashed lines denote the critical strains (ε_{cr}). c-f) Deformation sequences of building-block structures with n = 0 (c), n =1 (d), n = 2 (e), and n = 3 (f), respectively.

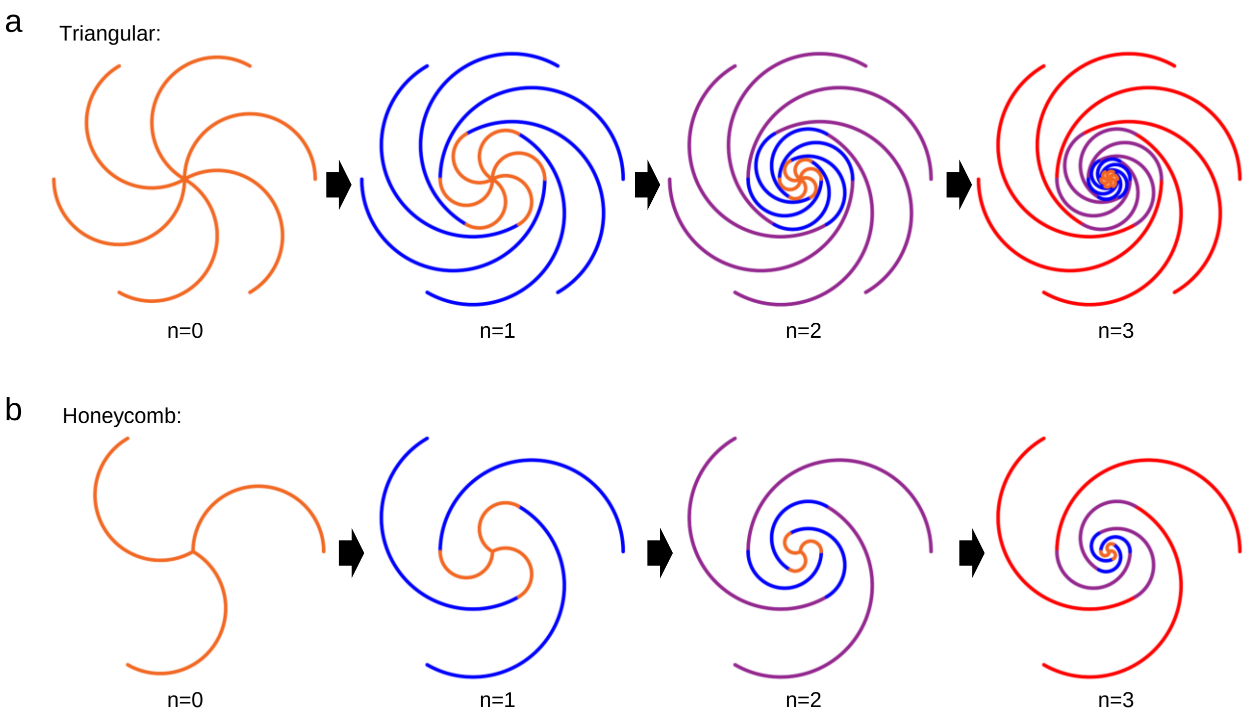

Figure S3. Schematic illustrations of unit-cell structures for triangular (a) and honeycomb (b) HSNMs with different hierarchical order (n = 0, 1, 2, 3).

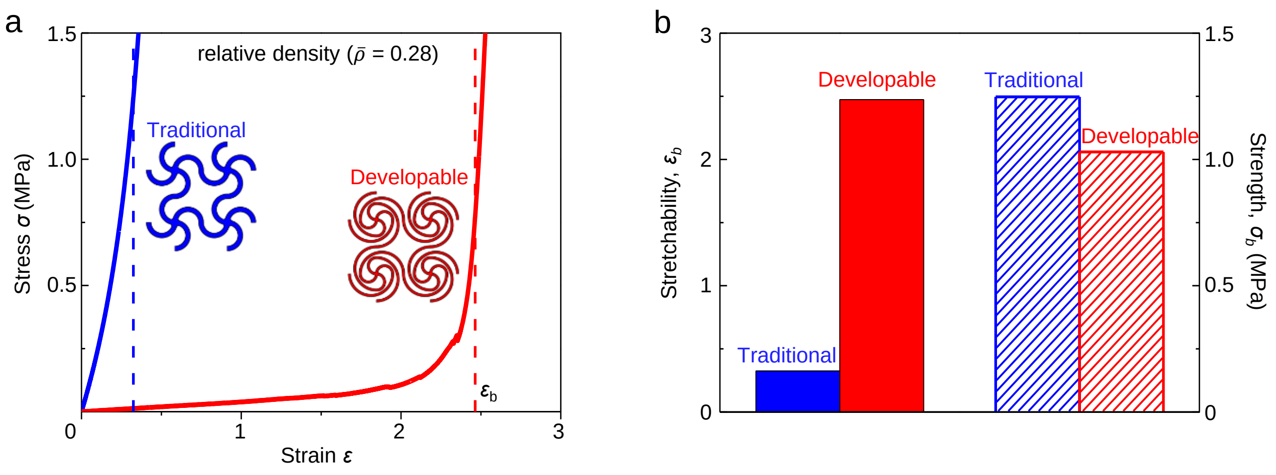

Figure S4. Comparison of the mechanical properties of proposed HSNMs and traditional SNMs with a same relative density ($\bar{\rho}$ = 0.28). a) FEA results of stress-strain curves for proposed HSNMs and traditional SNMs, based on the measured mechanical properties of 3D printed constituent materials. The insets present the schematic illustrations (2×2 unit cells) of these two network specimens. b) Predicted stretchability (ε_b) and strength (σ_b) for these two network specimens.

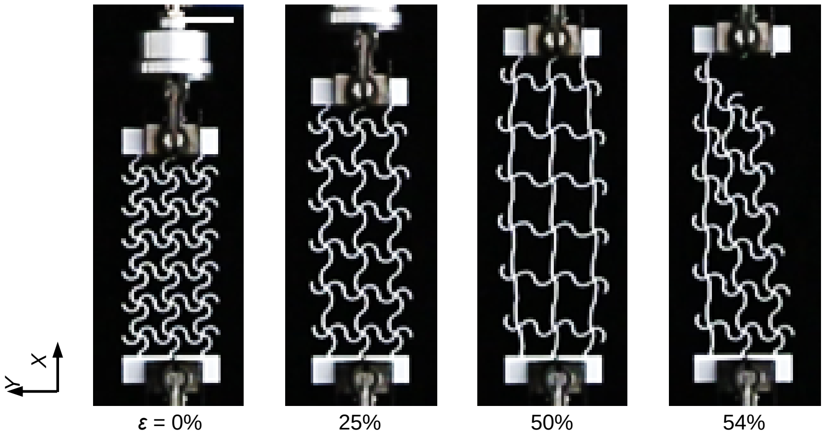

Figure S5. Optical images of deformation sequences for the traditional SNM specimens under uniaxial stretching.

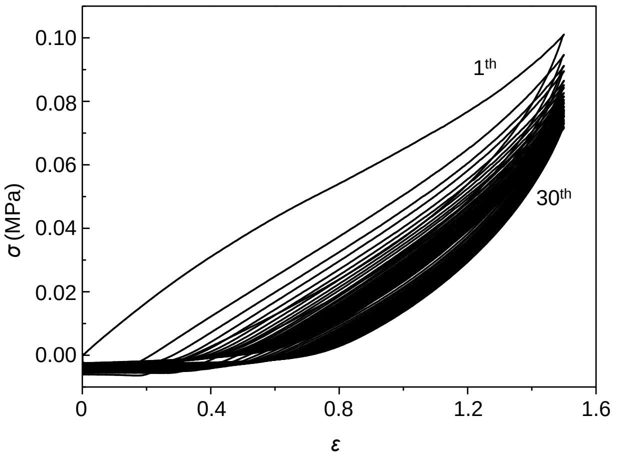

Figure S6. Cyclic stress-strain curves of HSNM specimen applied in Figure 2d-i.

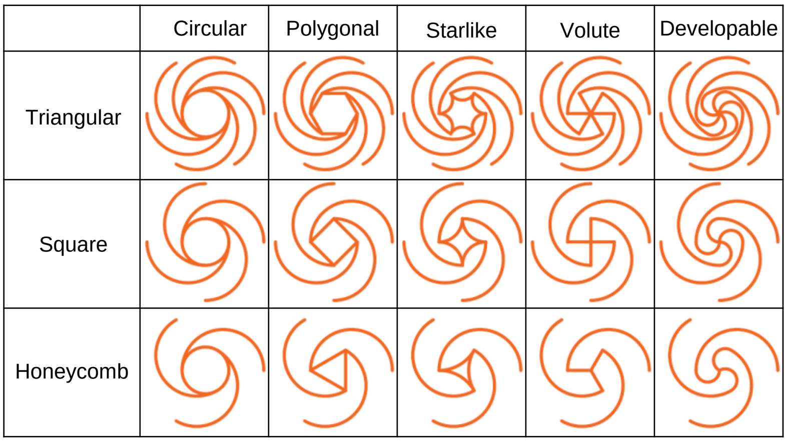

Figure S7. Schematic illustrations of unit-cell structures consisting of various lattice nodes and different lattice topologies.

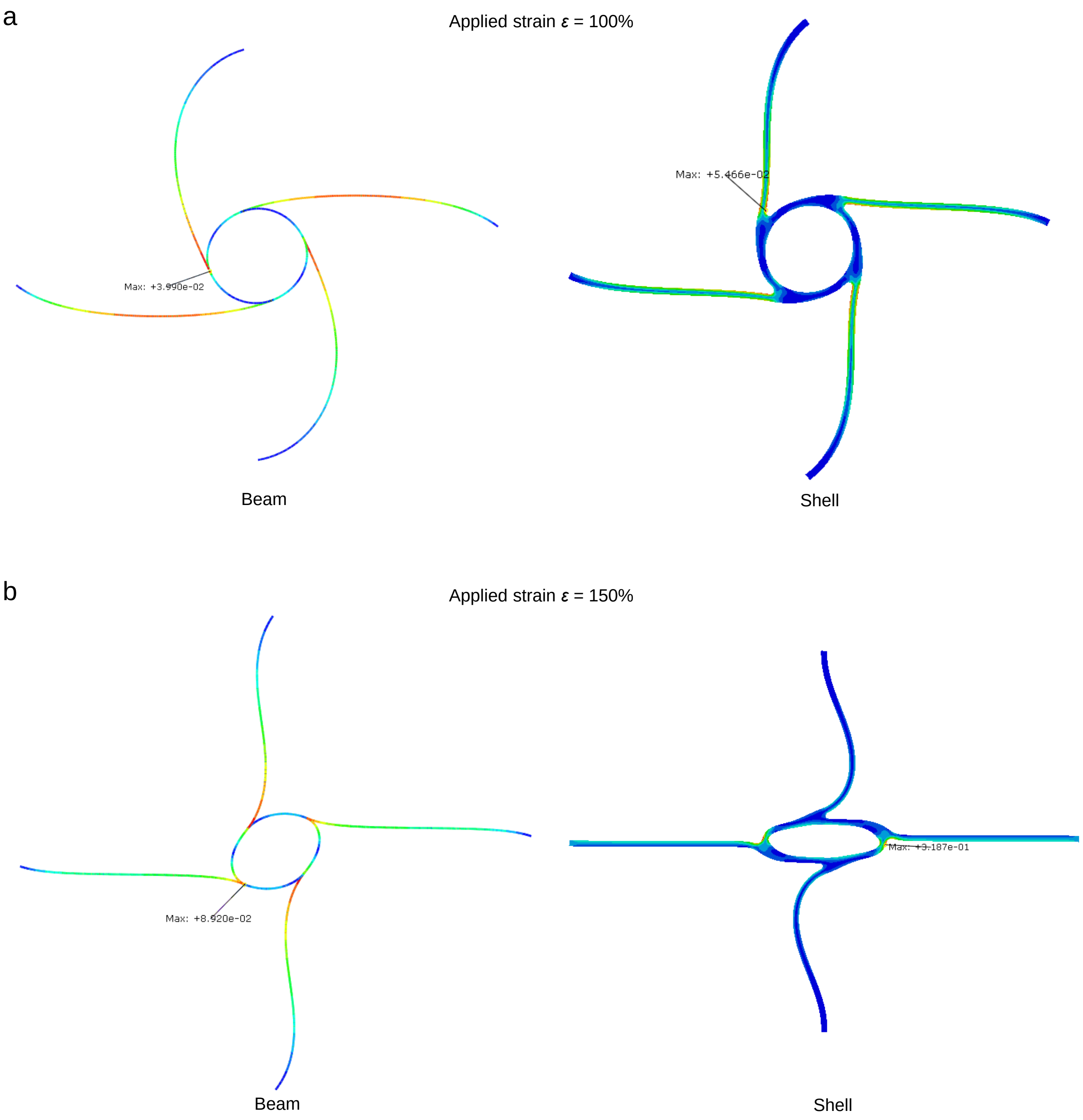

Figure S8. Beam-based FEA and shell-based FEA results of deformed configurations and ε_{max} distributions for a representative unit cell of SNMs with circular nodes, in the case of applied strain ε = 1.0 (a) and ε = 1.5 (b). Locations and values of the peak ε_{max} are marked in the figure for comparison.

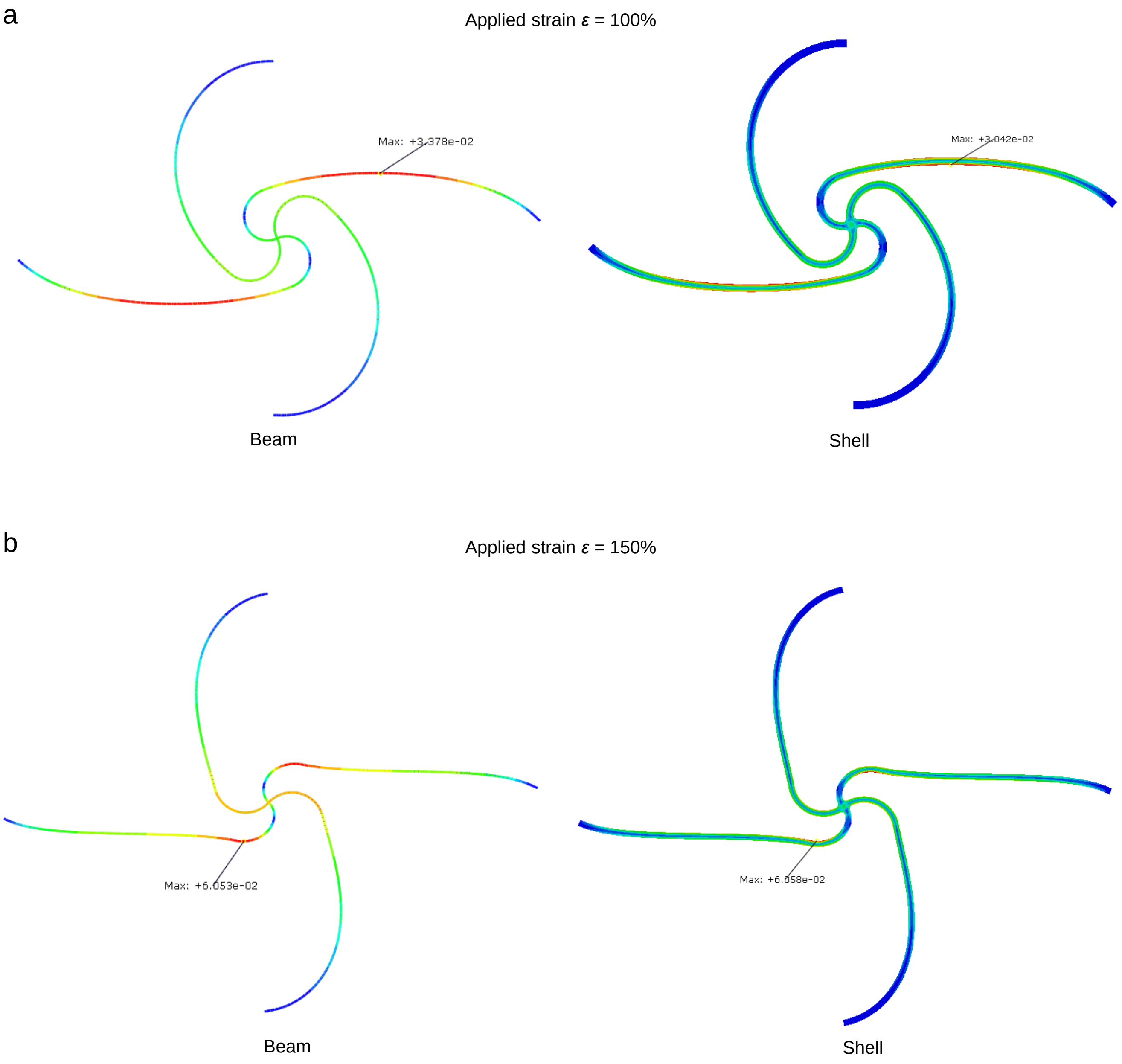

Figure S9. Beam-based FEA and shell-based FEA results of deformed configurations and ε_{max} distributions for a representative unit cell of HSNMs with developable nodes, in the case of applied strain ε = 1.0 (a) and ε = 1.5 (b). Locations and values of the peak ε_{max} are marked in the figure for comparison.

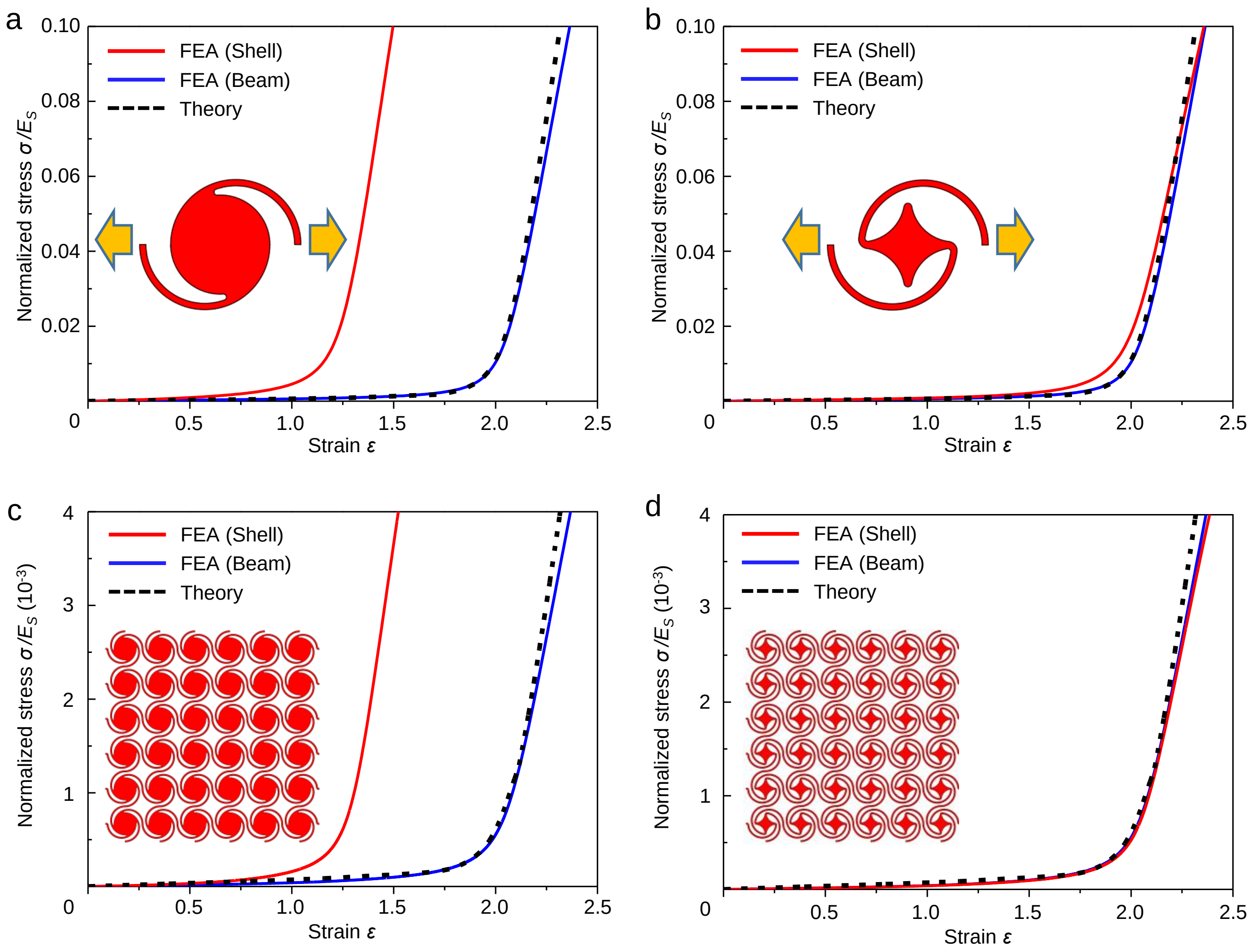

Figure S10. Theoretical prediction of normalized stress-strain curves for SNM with circular nodes and starlike nodes. a-b) Shell-based FEA and beam-based FEA results, as well as theoretical results for building-block structures with solid circular nodes (a) and solid starlike nodes (b). c-d) Similar results for network structures with solid circular nodes (c) and solid starlike nodes (d).

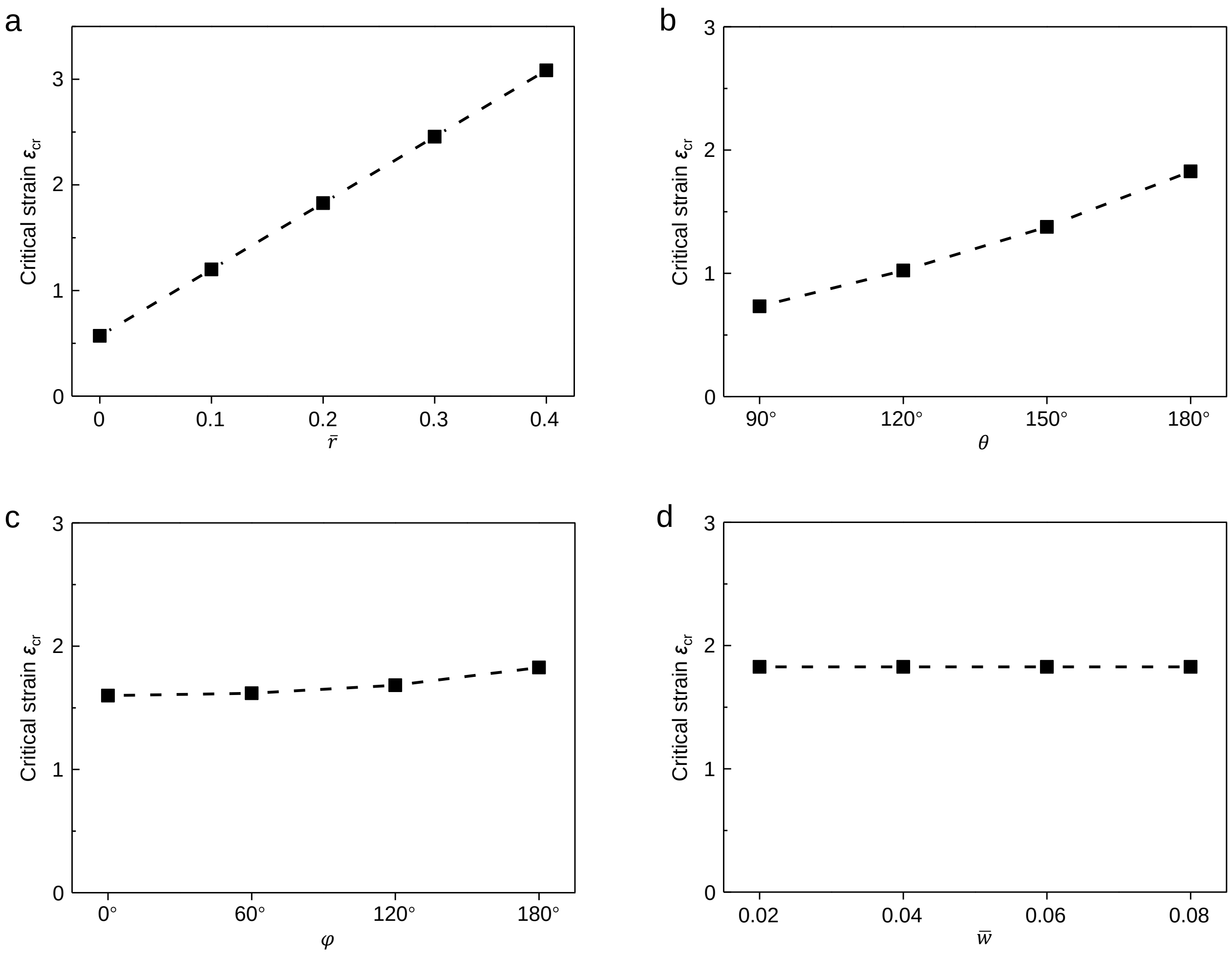

Figure S11. Effects of key geometric parameters on critical strains (ε_{cr}) of square HSNMs, including normalized radius $\bar{r}$ (a), joint angle θ (b), node angle φ (c), and normalized width $\bar{w}$ (d).

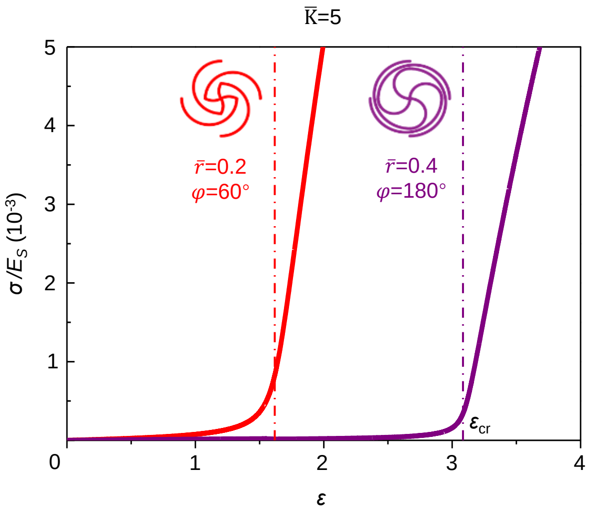

Figure S12. Normalized stress-strain curves for HSNMs with a same curvature ($\overline{K}$ = 5) but different sets of normalized radius ($\bar{r}$) and node angle (φ).

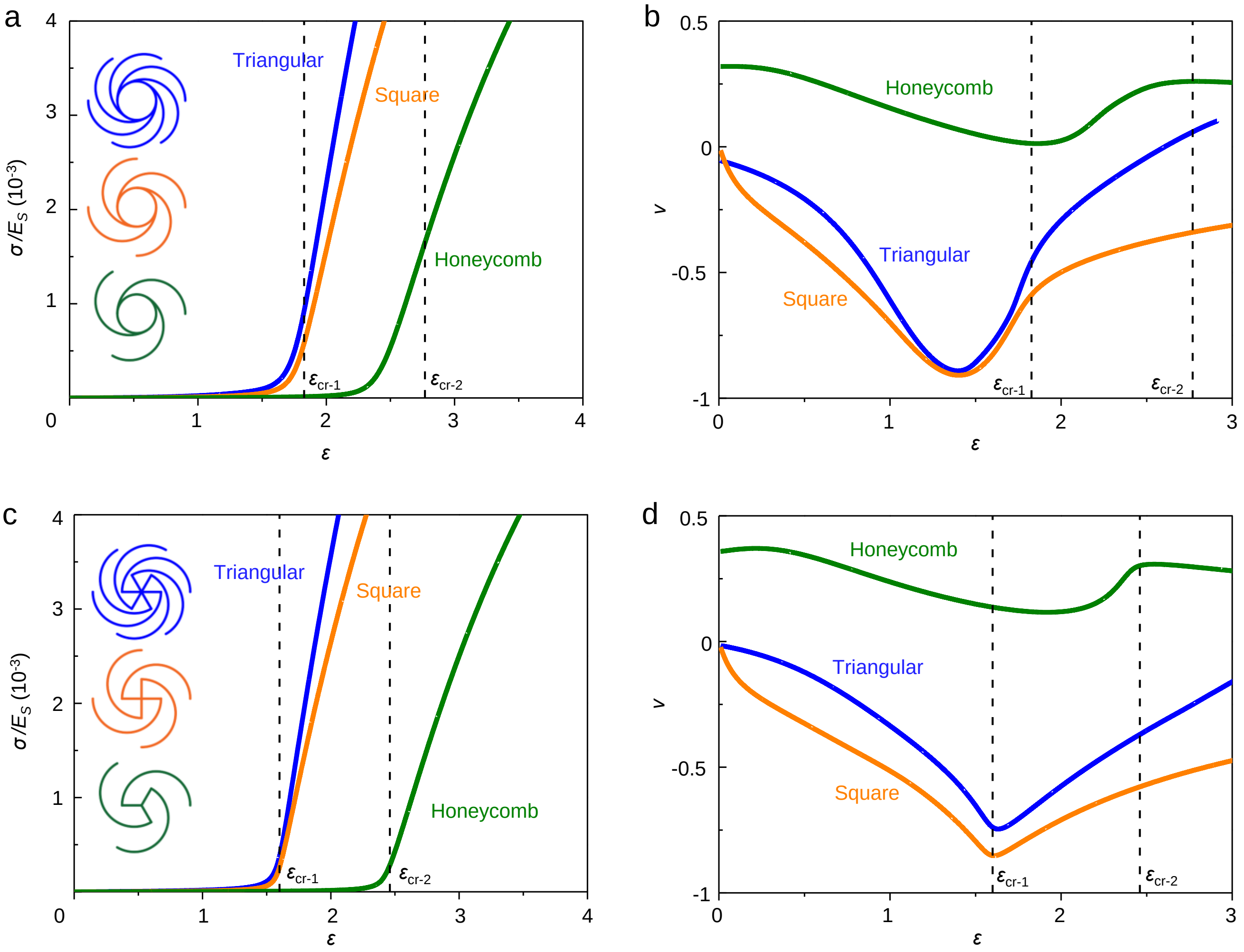

Figure S13. a-b) Effects of lattice topologies on stress-strain responses (a) and nonlinear Poisson's ratios (b) for the SNMs with circular nodes. c-d) Effects of lattice topologies on stress-strain responses (c) and nonlinear Poisson's ratios (d) for the SNMs with volute nodes. The dashed lines denote the critical strain ($\varepsilon_{cr\text{-}1}$) of SNMs with triangular and square topologies, as well as the critical strain ($\varepsilon_{cr\text{-}2}$) of honeycomb SNMs.

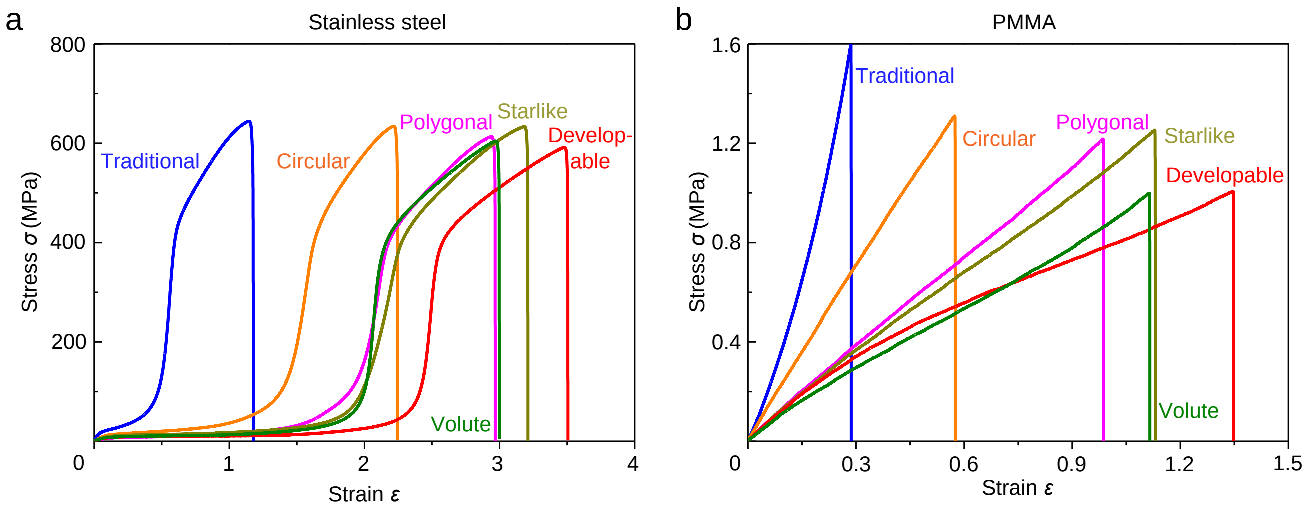

Figure S14. Experimental results of stress-strain curves for metallic (a) and PMMA-based (b) building-block specimens with different types of nodes
